# Catch & Release—rapid cost‐effective protein purification from plants using a DIY GFP‐Trap‐protease approach

**DOI:** 10.1111/tpj.70544

**Published:** 2025-11-12

**Authors:** Sebastian Schwartz, Carina Engstler, Susanne Mühlbauer, Eduard Windenbach, Tobias Wunder, Hans‐Henning Kunz, Benjamin Brandt

**Affiliations:** ^1^ Plant Biochemistry and Physiology LMU München Großhaderner Straße 2‐4 82152 Martinsried‐Planegg Germany

**Keywords:** plant protein isolation, FAST, fluorescent protein tag, dual‐fluorescent protein tag, low‐cost, modular cloning

## Abstract

The purification of proteins is the foundation to study their structure, function, biochemical properties, and interaction partners. In plant research, unique challenges arise from the complexity of plant tissues, interference of secondary metabolites, and sometimes the low abundance of target proteins. Many conventional plant protein purification methods rely on expensive reagents, multistep procedures, and labor‐intensive workflows, limiting their feasibility for many applications. Here, we present the ‘Catch & Release’ system, a cost‐effective, fast, and reliable one‐step purification workflow for the isolation of proteins from plant tissues. The Catch & Release toolbox includes a vector set, a homemade GFP‐Trap, and homemade proteases. We demonstrate how the Catch & Release vectors streamline cloning and transgenic plant selection through the fluorescence‐accumulating seed technology, which marks positive transformants with a strongly fluorescing seed coat. Each plasmid consists of four easy‐to‐exchange modules: a plant promoter, a cloning dropout marker, protease cleavage sites, and seven different epitope tags, including an innovative dual‐fluorescent tag, providing flexibility for diverse experimental needs. The *in vivo* functionality of all modules has been confirmed by complementation and transient protein expression experiments. We further show that besides facilitating standard molecular biological experimentation, fusion proteins expressed using our vector set in combination with homemade GFP‐Trap and proteases enable efficient and rapid isolation of enzymatically active, soluble, and high molecular weight membrane proteins directly from plants. By following our detailed reagent preparation instructions, purification costs can be decreased up to 60‐fold compared with commercially available options.

## Significance Statement

‘Catch & Release’ represents a cost‐effective, rapid, and reliable one‐step protein purification workflow that accelerates plant research. It offers a functionality‐confirmed and versatile binary vector set combining a variety of commonly used protein tags with fluorescent seed coat markers. We provide detailed instructions on producing DIY GFP‐Traps and proteases as well as the purification strategy. The combined system significantly reduces purification costs and time, enabling plant scientists to accelerate and advance plant structural biology and biochemistry.

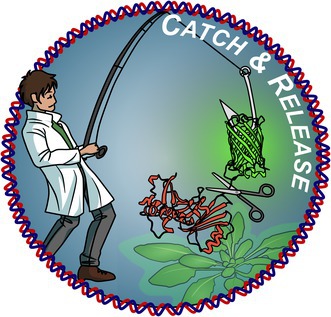

## INTRODUCTION

Understanding the function, structure, and regulation of proteins is fundamental to deciphering complex biological processes. However, as the biochemistry textbook states: ‘Never waste pure thoughts on an impure protein’ (Berg et al., 2019). Consequently, the ability to isolate and purify specific proteins in an active and native state is a cornerstone of plant molecular biology research (Amrhein et al., 2015). Purifying proteins from plant sources presents significant challenges compared with microbial or mammalian systems. The inherent complexity of plant tissues, often rich in cell walls, vacuoles, and interfering secondary metabolites (phenolics, pigments, polysaccharides), complicates extraction (Schillberg et al., 2019) and can compromise protein stability and activity (Du et al., 2022). Furthermore, many plant proteins of interest are expressed at low endogenous levels, requiring sensitive and efficient isolation strategies (Righetti & Boschetti, 2020).

The requirement for highly pure protein isolates is especially apparent in structural biology. Due to the above‐mentioned limitations, most plant protein expressions and isolations are carried out in heterologous systems, primarily *Escherichia coli*. In fact, 82% of high‐quality protein structures from Arabidopsis proteins found in the protein data bank (PDB; https://www.rcsb.org; see Table S4; Materials and Methods) were obtained using *E. coli*‐expressed proteins. However, heterologous expression of plant proteins, especially in prokaryotes, has considerable limitations such as the absence of eukaryotic post‐translational modifications (PTMs) (Zhang et al., 2022), the lack of specific plant chaperones or cofactors, and a non‐native intracellular redox state (Bhatwa et al., 2021), all of which can affect correct folding. Improperly folded proteins may yield structural or functional data not accurately reflecting the native state. These missing factors, in combination with disparities in codon usage affecting translation fidelity and speed (Gustafsson et al., 2004), also frequently result in poor expression of plant proteins in prokaryotes. To overcome these limitations, the expression of plant proteins in their native expression host is favorable.

Conventional protein purification workflows often rely on time‐consuming, multistep chromatographic procedures, using expensive commercial affinity resins and reagents. Affinity tagging strategies (e.g., His‐tag or Strep‐tag) are commonly used for rapid one‐step isolation of recombinant proteins from many expression systems. However, their usability in plants is limited. This is in part due to the generation of stable transgenic lines expressing tagged proteins, which, depending on the species, can be a time‐consuming process (Clough & Bent, 1998; Gao, 2021). Another reason is secondary metabolites interference with the tag‐resin binding (e.g., biotin for Strep‐tag, negatively charged molecules for IMAC purifications, or phenolic compounds in general). These limitations are demonstrated by only 0.6% of Arabidopsis protein structures deposited in the PDB originating from proteins isolated from Arabidopsis. All protein isolations from Arabidopsis giving rise to structures are complex and use multiple steps (see Table S4; Materials and Methods). These combined factors—tissue complexity, low abundance, and no suitable one‐step affinity tags resulting in lengthy workflows—limit the throughput, scalability, and accessibility of protein purification from plants.

Advanced modular cloning systems, such as those based on Golden Gate assembly utilizing Type IIS restriction enzymes (e.g., BsaI, BpiI), have been developed for the construction of complex expression vectors (Engler et al., 2008). These systems enable the ordered assembly of multiple DNA fragments (promoters, coding sequences, tags, terminators) in a single reaction which can be subsequently assembled into multiexpression unit constructs in a second reaction. However, establishing and utilizing Golden Gate or related plant‐specific systems like GreenGate (Lampropoulos et al., 2013) requires a significant upfront investment including creating or acquiring libraries of standardized and compatible DNA modules. During the creation of sequence‐verified entry modules, the DNA sequences must be free of the specific Type IIS recognition sites used for the subsequent assemblies. This can be challenging, especially for large or complex DNA fragments such as genomic plant DNA fragments. While powerful for multiexpression unit constructs, the complexity and setup effort associated with these systems might be less justified for the routine generation of simpler vectors commonly used for expression and purification of tagged proteins.

To resolve current limitations for the purification of plant proteins in their native hosts, we developed and combined publicly available resources into the ‘Catch & Release’ system, a versatile and cost‐effective toolbox designed to enable efficient protein expression and purification of plant proteins. This system integrates several key features: (i) efficient vector construction using multiple cloning strategies facilitated by a visual dropout marker; (ii) accelerated generation of stable transgenic lines through integration of fluorescence‐accumulating seed technology (FAST; Shimada et al., 2010); (iii) a set of seven interchangeable C‐terminal tags, including standard small peptide tags, fluorescent proteins, and innovative dual‐fluorescent tags, providing flexibility for detection, localization, and purification; (iv) fluorescent tags in combination with protease cleavage sites (TEV or HRV 3C) are compatible with homemade proteases and a DIY GFP‐Trap to enable cheap and rapid purification of native target proteins expressed in plants with a single isolation step (one‐step purification).

In this work, we demonstrate the utility and robustness of the Catch & Release toolbox for both transiently (*Nicotiana benthamiana*) and stably (*Arabidopsis thaliana*) expressed plant proteins. We show its successful application for the functional complementation, correct subcellular localization, and efficient purification of POTASSIUM CATION EFFLUX ANTIPORTER 1 (KEA1), a ~123 kDa large, chloroplast inner envelope membrane protein. Furthermore, we validate the system for purifying the soluble stromal enzyme PHOSPHOGLYCERATE DEHYDROGENASE 3 (PGDH3) from transient expression in *N. benthamiana* as intact (dimeric) and enzymatically active protein using the novel dual‐fluorescent tag.

## RESULTS AND DISCUSSION

### Catch & Release includes a flexible vector set for cloning and protein analysis

**Figure 1:**
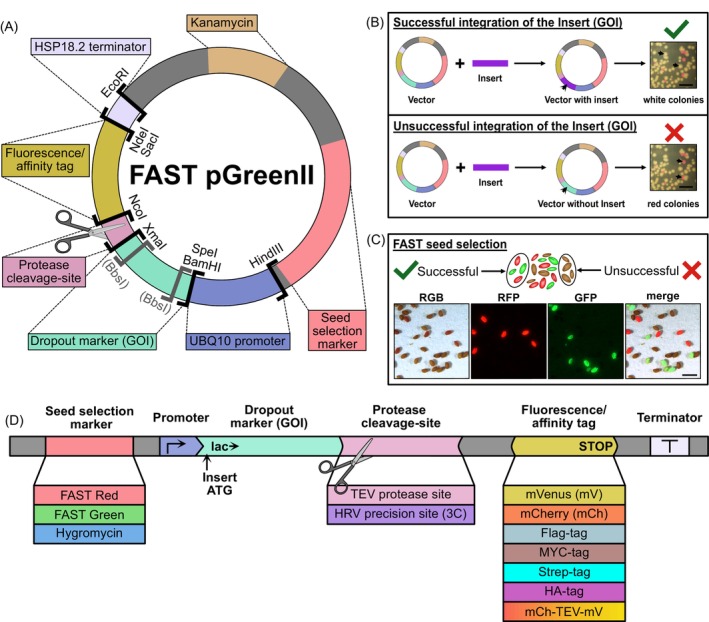
Overview and features of the Catch & Release vector set. (A) General design of the Catch & Release binary vectors highlighting key restriction sites, with (gray) sites indicating those removed upon successful Golden Gate assembly. (B) Red/white colony screening for successful DNA incorporation via Golden Gate‐, Gibson‐ or classical restriction cloning. Scale bar = 0.5 cm. (C) Seeds from Arabidopsis plants individually transformed with either FAST‐Green or FAST‐Red vectors, pooled and examined under a fluorescence stereoscope. Strongly fluorescent seeds were selected for propagation to obtain homozygous T_3_ lines. Scale bar = 1 mm. (D) Catch & Release toolbox comprising 13 parts, categorized into four subtypes with a total of 30 vectors (listed in Table 1). Vectors incorporating the HRV 3C precision site are available exclusively with the mVenus tag and the dual‐fluorescent tag. All expression vectors include a stop codon after the respective C‐terminal tags but not the initiator start codon.

To overcome common bottlenecks in plant transformation and functional protein studies, we established the ‘Catch & Release’ toolbox (Figure 1). The binary vector system included in the toolbox leverages a modular vector design, accelerating construct generation, simplifying transgenic line selection, and providing flexibility for downstream protein characterization.

‘Catch & Release’ plasmids offer cloning versatility, since they are fully compatible with most common methods such as GoldenGate assembly (Engler et al., 2008), Gibson/InFusion (Gibson et al., 2009) assembly, SLIC (Jeong et al., 2012), and traditional restriction–ligation cloning. To facilitate these methods, we provide a detailed sequence map of the multiple cloning sites, which specifies potential cloning scars and minimal amino acid linkers and overhangs that remain on the target protein after cleavage with TEV or 3C protease (Figure S1A). A cloning guide is also included, outlining recommended primer overhangs (Figure S1B) and presenting cloning efficiencies (Figure S1C). For efficient screening, the vectors contain a visual dropout marker cassette under the control of a *lac* promoter, driving the constitutive expression of a red fluorescent protein (RFP) gene (Figure 1A–B). This feature enables the identification of recombinant clones directly on the KANAMYCIN supplemented selection plate (Figure 1B). Colonies with successfully incorporated DNA insert lose RFP expression and appear white. In contrast, non‐recombinant background colonies remain red. This visual screening streamlines cloning by reducing laborious PCR or digestion checks.

To accelerate the generation of homozygous transgenic plant lines, a major time constraint in plant research, we incorporated the FAST (Shimada et al., 2010) into the vector set. The FAST system enables non‐invasive selection of transformed seeds based on seed coat fluorescence, eliminating the need for antibiotic or herbicide resistance markers that can impair plant development (Yin et al., 2008) and trigger pleiotropic phenotypic alterations. Additionally, using fluorescent seed coats as transgenic markers results in major time savings during segregation analyses and establishment of homozygous plant lines. Our toolbox includes both FAST‐Red and FAST‐Green options. Floral dip *Agrobacterium*‐assisted transformation (Clough & Bent, 1998) of Arabidopsis plants had a transformation efficiency of 0.5%, as determined by fluorescence‐based seed counting.

The FAST system provides a highly efficient and rapid method for selecting transgenic lines, achieving over 92% of selected plants carrying the transgene, which is an increase from the 78% observed with traditional HYGROMYCIN screening. The combination of FAST‐Red and FAST‐Green cassettes also enables the selection for plants carrying multiple transgenes (Figure 1C for individually transformed and pooled seeds; Ursache et al., 2021). To ensure broad experimental versatility, the toolkit also includes conventional HYGROMYCIN selection vectors. All selection markers included in the vector set work side‐by‐side with the most common selection markers used in the major seed collections (BASTA, KANAMYCIN, or SULFADIAZINE).

A cornerstone advantage of the Catch & Release toolbox lies in its variable protein tagging strategy, which offers a suite of seven distinct C‐terminal tags, encompassing small peptide tags (e.g., Strep, Flag, MYC, HA) for detection and isolation and single fluorescent proteins (e.g., mVenus, mCherry) for localization, isolation, and detection (Figure 1D).

In addition, we designed a novel dual‐fluorescent tag, which is offered in two configurations that differ in mCherry's removability: mCherry‐TEV‐mVenus (non‐removable mCherry) and 3C‐mCherry‐TEV‐mVenus (fully removable mCherry). Critically, each tag is preceded by protease cleavage sites (TEV or HRV 3C) engineered to ensure tag removal, leaving only a minimal number of amino acid residues behind on the target protein. Removable tags permit initial purification/detection followed by controlled cleavage *in vitro* to release the native protein. This approach ensures the suitability of the protein for further downstream functional and structural analyses by preventing potential interference with the fusion tag.

The system's modularity extends beyond tags. The set comprises 13 exchangeable components, yielding 30 ready‐to‐use binary vectors (Table 1). Importantly, key functional elements like protease sites, promoters, and terminators (e.g., the commonly used UBQ10 promoter and HSP18.2 terminator; Pratt et al., 2020) can be readily exchanged using designated restriction sites (Figure 1A). This adaptability empowers researchers to easily customize vectors for specific expression requirements such as tissue‐specific expression or usability in different plant species.

**Table 1:** Catch & release vector set including Addgene IDs

Construct	Addgene ID; European Plasmid Repository (EPR) ID
pG20_Hyg	240650; 983
pG20_TEV_mVenus_Hyg	240651; 984
pG20_TEV_mCherry_Hyg	240652; 985
pG20_mCherry_TEV_mVenus_Hyg	240653; 986
pG20_3C_mCherry_TEV_mVenus_Hyg	240654; 987
pG20_TEV_2xStrepII_Hyg	240655; 988
pG20_TEV_MYC_Hyg	240656; 989
pG20_TEV_HA_Hyg	240657; 990
pG20_TEV_Flag_Hyg	240658; 991
pG20_3C_mVenus_Hyg	240659; 992
pG20_FR	240660; 993
pG20_TEV_mVenus_FR	240661; 994
pG20_TEV_mCherry_FR	240662; 995
pG20_mCherry_TEV_mVenus_FR	240663; 996
pG20_3C_mCherry_TEV_mVenus_FR	240664; 997
pG20_TEV_2xStrepII_FR	240665; 998
pG20_TEV_MYC_FR	240666; 999
pG20_TEV_HA_FR	240667; 1000
pG20_TEV_Flag_FR	240668; 1001
pG20_3C_mVenus_FR	240669; 1002
pG20_FG	240670; 1003
pG20_TEV_mVenus_FG	240671; 1004
pG20_TEV_mCherry_FG	240672; 1005
pG20_mCherry_TEV_mVenus_FG	240673; 1006
pG20_3C_mCherry_TEV_mVenus_FG	240674; 1007
pG20_TEV_2xStrepII_FG	240675; 1008
pG20_TEV_MYC_FG	240676; 1009
pG20_TEV_HA_FG	240677; 1010
pG20_TEV_Flag_FG	240678; 1011
pG20_3C_mVenus_FG	240679; 1012

### Functional validation of Catch & Release constructs in transgenic plants

**Figure 2:**
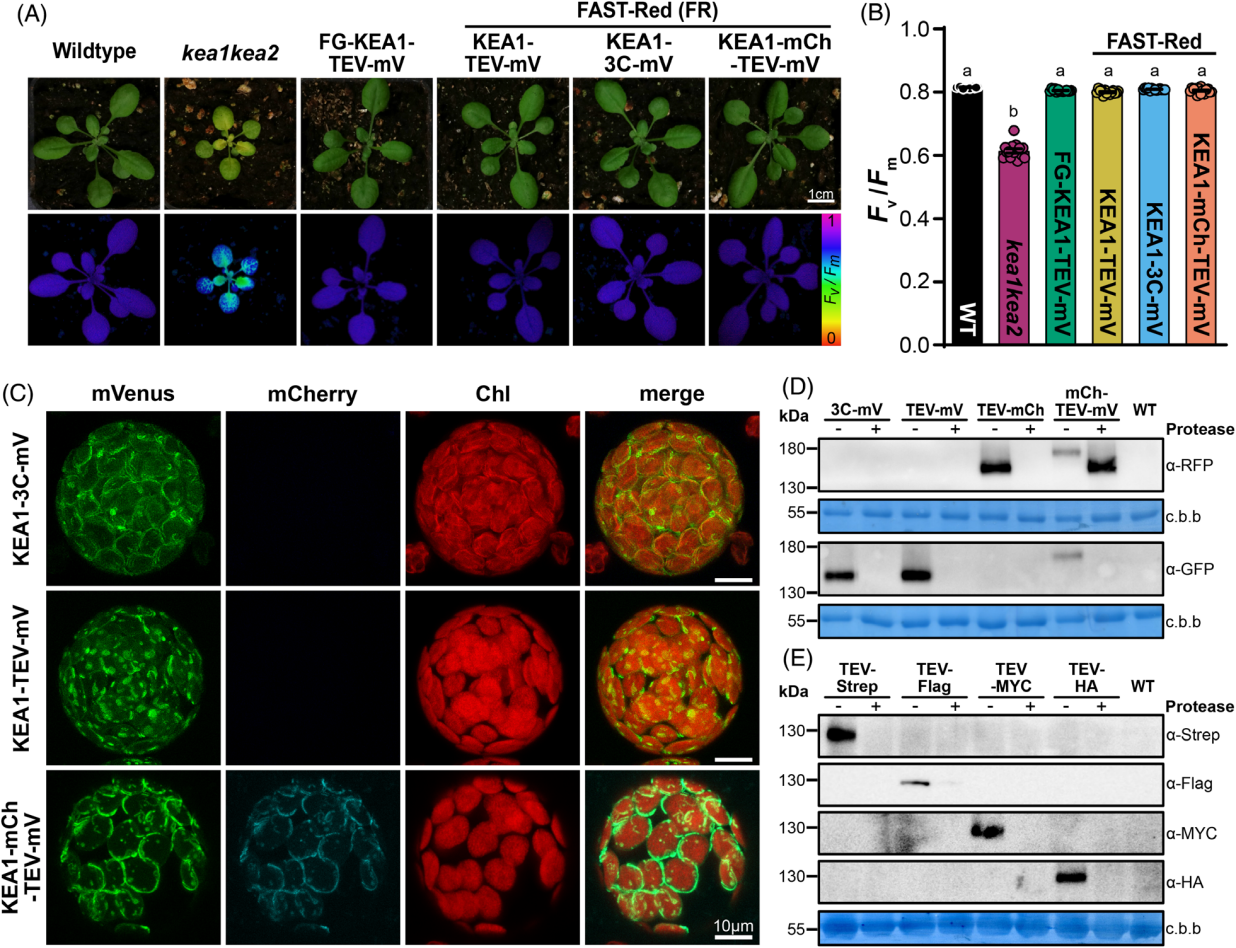
Functional characterization of KEA1 constructs using Catch & Release vectors. (A) Photographs and PAM images (*F*_v_/*F*_m_) comparing WT, *kea1‐1kea2‐1*, and complemented *kea1‐1kea2‐1* lines expressing FAST‐Green (FG)‐KEA1‐TEV‐mVenus, FAST‐Red (FR)‐KEA1‐TEV‐mVenus, FAST‐Red‐KEA1‐3C‐mVenus, and FAST‐Red‐KEA1‐mCherry‐TEV‐mVenus using a UBQ10 promoter. Scale bar = 1 cm. (B) *F*_v_/*F*_m_ in WT, *kea1‐1kea2‐1*, and complemented plants expressing FAST‐Green (FG)‐KEA1‐TEV‐mVenus, FAST‐Red‐KEA1‐TEV‐mVenus, FAST‐Red‐KEA1‐3C‐mVenus, and FAST‐Red‐KEA1‐mCherry‐TEV‐mVenus. Data are presented as mean ± SEM (*n* = 14). Statistical significance was determined via one‐way anova, with different letters indicating significantly different groups. (C) Confocal laser micrographs of Arabidopsis protoplasts expressing KEA1‐TEV‐mVenus, KEA1‐3C‐mVenus, and KEA1‐mCherry‐TEV‐mVenus. Fluorescence images show KEA1‐mVenus (green, left), KEA1‐mCherry (cyan, middle left), chlorophyll autofluorescence (red, middle right), and a merged view (right). Scale bar = 10 μm. (D) Immunoblot analysis of protease site accessibility in whole‐leaf extracts from WT and complemented *kea1kea2* plants expressing KEA1‐3C‐mVenus, KEA1‐TEV‐mVenus, KEA1‐TEV‐mCherry, and KEA1‐mCherry‐TEV‐mVenus. The same membrane was stained with Coomassie brilliant blue (c.b.b.) and rubisco large subunit (rbcL) was used as a loading control. The exact same protein extracts were used for pre‐ and post‐protease treatment samples. (E) Immunoblot analysis of protease site accessibility in *Nicotiana benthamiana* leaves infiltrated with KEA1‐TEV‐Strep, KEA1‐TEV‐Flag, KEA1‐TEV‐MYC, and KEA1‐TEV‐HA. Un‐infiltrated leaves served as WT controls. The same membrane was stained with Coomassie brilliant blue (c.b.b.) and rbcL was used as a loading control. The exact same proteins extracts were used for pre‐ and post‐protease treatment samples.

To validate the functionality of proteins expressed using our vectors, we used our most bulky tags to assess their risk of interference with protein function. Here, we performed complementation studies in the *Arabidopsis thaliana kea1‐1kea2‐1* double mutant (Kunz et al., 2014). This loss‐of‐function mutant lacks the plastid inner envelope cation efflux antiporters KEA1/KEA2, which results in delayed chloroplast development and impaired photosystem II quantum yield (*F*_v_ */F*_m_) in younger leaves (Figure 2A,B; DeTar et al., 2021).

We generated transgenic lines expressing KEA1 fused to C‐terminal mVenus (via TEV or HRV 3C linker) or the dual mCherry‐TEV‐mVenus tag. Homozygous third generation progeny (T_3_) were selected efficiently using integrated FAST markers. Pulse‐modulated chlorophyll fluorescence measurements revealed that the reduced *F*_v_ */F*_m_ phenotype of the *kea1‐1kea2‐1* mutant (0.58 ± 0.03) was fully restored to wild‐type levels (Col‐0: 0.81 ± 0.01) in all complemented lines expressing tagged KEA1 variants (e.g., KEA1‐TEV‐mVenus: 0.81 ± 0.01; Figure 2A,B).

Next, we examined the localization of the tagged KEA1 proteins in protoplasts isolated from the complemented Arabidopsis lines. Confocal laser microscopy revealed that fluorescence signals from mVenus, or mCherry and mVenus (in dual‐tagged lines) were localized to the periphery of chloroplasts (Figure 2C), consistent with the known chloroplast envelope localization of KEA1 (Bölter et al., 2020). While the fluorescence signal was concentrated at the chloroplast periphery, we also observed patchy protein accumulation, particularly in the protoplasts expressing dual‐tagged KEA1 and the TEV‐mVenus line. A similar phenomenon has been reported for envelope localized AtKEA2‐GFP (Aranda‐Sicilia et al., 2016). Differences in the apparent localization between the different KEA1 complementation constructs may be a consequence of varying transgene expression strengths, differences in cell age of the analyzed protoplasts, or artifacts from the protoplast isolation procedure. However, the precise co‐localization of mCherry and mVenus signals in the dual‐tagged construct further confirmed the integrity and proper trafficking of the full‐length fusion protein, demonstrating the system's reliability for accurate subcellular localization studies, even with large fusion tags such as the mCherry‐TEV‐mVenus (54.7 kDa).

These results demonstrate that KEA1 proteins tagged using the Catch & Release toolbox are correctly expressed, folded, localized, and biologically active in planta, capable of rescuing physiological impairments. The successful application to KEA1, which presents considerable challenges due to its size, import into plastids, and targeting to the inner envelope membrane, strongly suggests the system's applicability to proteins with less complex folding or targeting. It also validates the system's suitability for functional genetic studies.

We next aimed to confirm the accessibility of the protease cleavage sites, a central design principle. We tested protease‐assisted removal of the fluorescence tag fused to KEA1 by immunoblotting of total protein extracts from the stably transformed Arabidopsis lines (Figure 2D). Antibodies against GFP (also detecting mVenus) and mCherry recognized proteins of the molecular weights consistent with the expected size of the full‐length KEA1 fusion proteins (~150 kDa for single tags, ~175 kDa for the dual‐tag). TEV and HRV 3C proteases were produced in‐house using publicly available expression vectors (Figure S2A–D; see Materials and Methods). Upon incubation of the extract with the appropriate protease (TEV or HRV 3C) for up to 4 h, no immune signal for full‐length KEA1 fluorescent fusion proteins was detected, indicative of protease‐assisted removal of the tag. Treatment of the dual‐tagged KEA1‐mCherry‐TEV‐mVenus protein extract with TEV protease resulted in a clear shift in the mCherry signal's apparent molecular weight and loss of the mVenus immunoblotting signal, confirming precise cleavage at the engineered protease site.

We also tested the expression, tag accessibility, and removal using small peptide‐tagged KEA1 constructs transiently expressed in *N. benthamiana* leaves (Figure 2E), a widely used system for transient protein expression in plants. Immunoblotting detected proteins around 130 kDa, consistent with the expected size of KEA1 (~123 kDa; Bölter et al., 2020) plus the small peptide tags (e.g., Strep, Flag, MYC, HA). Subsequent treatment with TEV protease removed these tags as apparent by the loss of the respective tag detection, confirming efficient tag cleavage in this transient *in planta* expression system.

These results not only validate the functionality of the small peptide tags and proteases‐assisted tag removal but also showcase the Catch & Release toolbox's adaptability across different plant species and expression strategies (stable versus transient).

### Workflow for the isolation of native KEA1

**Figure 3:**
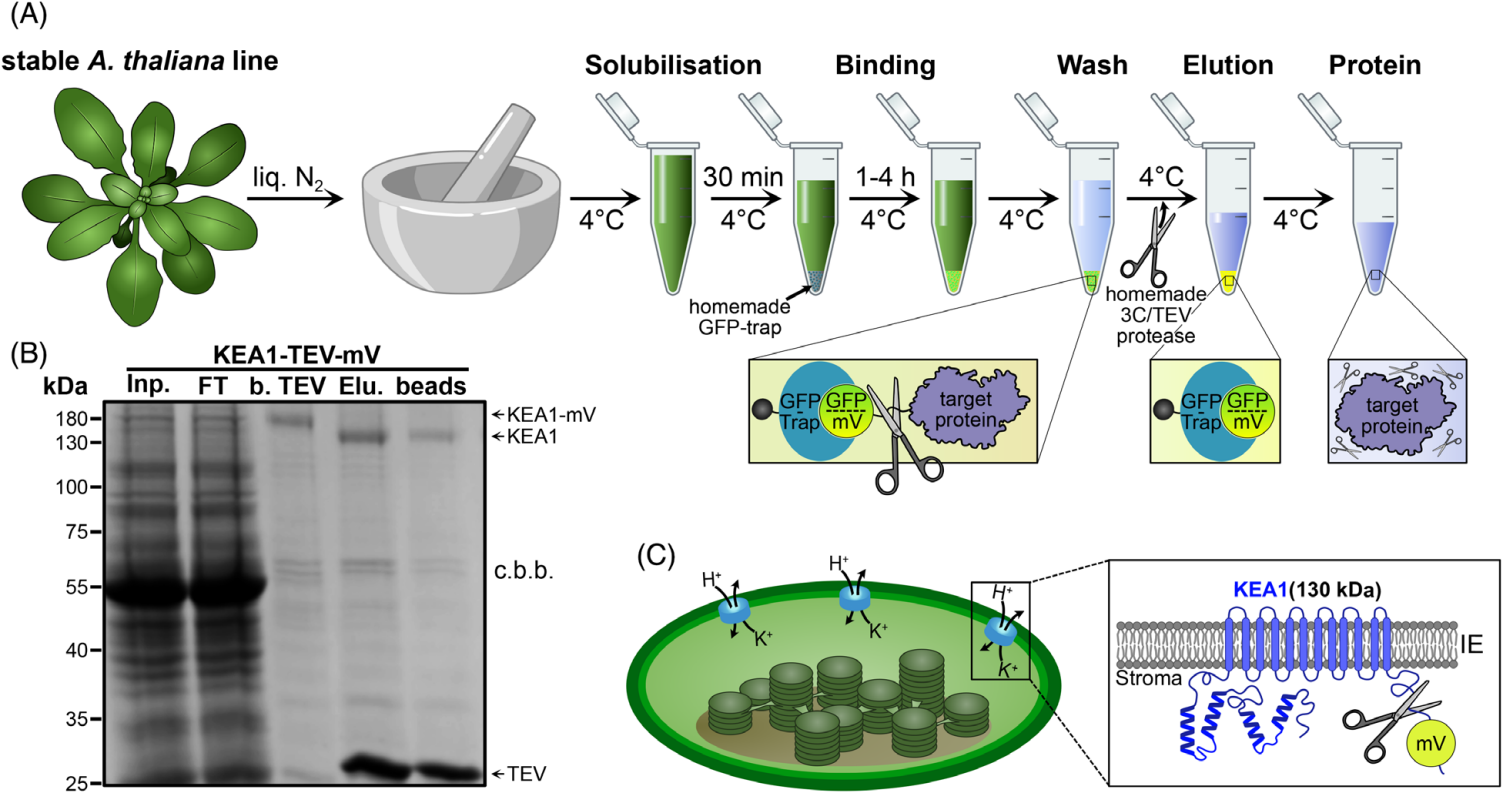
Isolation and structural features of KEA1 from *Arabidopsis thaliana*. (A) Schematic overview of the protein purification workflow using the homemade GFP‐Trap and proteases. *Arabidopsis thaliana* plants were first ground to powder in liquid nitrogen and solubilized for 30 min. The solubilized protein mixture was then incubated with homemade GFP‐Trap for 1–4 h at 4°C, followed by multiple wash steps. The protein was released by on‐resin protease digestion, resulting in an elution containing both the non‐tagged target protein and the protease. For information on volumes please refer to the Material and Methods section. (B) SDS‐PAGE analysis verifying successful KEA1 purification from stable *A. thaliana* lines, using the described workflow. Lanes represent: Total protein lysate (Input, Inp.), unbound fraction after incubation with GFP‐Trap (Flow‐Through, FT), affinity‐bound protein before protease cleavage (b. TEV), protein eluted after TEV cleavage (Elution, Elu.), and the GFP‐Trap resin after elution (beads). C.b.b. indicates that the gel was stained with Coomassie brilliant blue. (C) Schematic representation of the plastid inner envelope potassium cation efflux antiporter KEA1. The N‐terminal region is predicted to contain 2–3 coiled‐coil domains (depicted as helical structures), while the C‐terminal domain, facing the stroma, is fluorescently tagged and includes the KTN domain.

The successful phenotypic complementation of the *kea1‐1kea2‐1* double mutant by the KEA1‐TEV‐mVenus construct served as the initial validation, confirming that C‐terminal tagging with TEV‐cleavable mVenus did not compromise the function or correct subcellular localization of the KEA1 transporter (Figure 2A,C). This prompted us to use these lines for the purification of the large multimembrane spanning plastid inner envelope protein KEA1. The presence of the TEV protease cleavage site engineered between KEA1 and the mVenus tag allows for specific affinity capture via GFP‐Trap followed by its precise removal to yield native protein (Figure 3A).

Building upon the validated KEA1‐TEV‐mVenus line, we developed and implemented a cost‐effective and scalable one‐step affinity purification strategy (Figure 3A). Purifying KEA1, an integral membrane protein with up to 12 predicted transmembrane domains localized to the plastid inner envelope, presents significant biochemical challenges. Topological predictions place the N‐terminus, containing putative coiled‐coil domains, and the C‐terminus, featuring the conserved KTN (K^+^ transport, nucleotide‐binding; Roosild et al., 2002) module, within the plastid stroma (Figure 3C). The workflow centers on the use of a homemade GFP‐Trap affinity resin. The GFP‐binding DARPin of the homemade GFP‐Trap binds to GFP, YFP, CFP, and their derivates (i.e., mVenus) with high affinity while no interaction could be detected with mCherry or mRuby (Hansen et al., 2017).

We produced the GFP‐Trap affinity resin with a binding capacity of 0.8 mg GFP ml^−1^ resin in‐house using a slightly adapted purification and coupling protocol (Figure S3A; see Materials and Methods for details). The resulting resin proved highly stable when stored at 4°C, remaining functional for at least 2 years (Figure S3B). The DIY GFP‐Trap can be produced within 3 days and offers a significant cost reduction, currently saving up to 30 000 Euro compared with purchasing a commercially equivalent amount of resin (Figure S3C). Combined with in‐house produced TEV or HRV 3C proteases (Figure S2A–D), this approach substantially lowers the financial barrier for protein purifications. A standard purification reaction from plant tissue (40 μl of GFP‐Trap slurry, 30 μg of protease) is currently estimated to cost between 0.75€ and 1.25€, translating into a cost reduction of up to 60‐fold compared with commercially available alternatives (Figures S2F and S3C).

The protocol commenced with cryogenic tissue disruption to preserve protein integrity, followed by solubilization (Figure 3A). After removal of insoluble components, incubation with the GFP‐Trap resin under controlled conditions (4°C) facilitated efficient capture of the KEA1‐TEV‐mVenus fusion protein. SDS‐PAGE analysis confirmed the isolation of the full‐length KEA1‐TEV‐mVenus fusion protein, which migrated at an apparent molecular weight of approximately 150 kDa (Figure 3B). Due to the high affinity between the GFP‐Trap‐binding moiety and the mVenus tag, competitive elution is ineffective, for example, with free GFP. Therefore, we employed native elution via on‐resin proteolytic cleavage (e.g., with TEV protease), which specifically releases the target protein into the supernatant. This strategy retains the mVenus tag and potential non‐specifically bound proteins attached to the resin, yielding high‐purity, non‐tagged KEA1 in the supernatant (Figure 3A,B). Following on‐column incubation with TEV protease, the eluted KEA1 protein exhibited a distinct shift in mobility to approximately 130 kDa. The observed decrease in molecular weight corresponds to the expected size of the mVenus tag (~27 kDa), confirming the specific proteolytic cleavage at the TEV site (Figure 3B). This workflow yielded >1.5 μg of native, tag‐free, and pure KEA1 protein per gram of starting material (ground plant tissue).

Our results demonstrate the robustness of this cost‐efficient workflow, yielding high‐purity plant proteins for biochemical characterizations, such as structural elucidations, enzymatic activities, or immunoprecipitation (IP) experiments.

### Transiently expressed PGDH3 purification via dual‐fluorescence tag system

We next evaluated the utility of the novel dual‐fluorescence tag vector system (mCherry‐TEV‐mVenus) for protein production and purification from other plant sources via transient expression in *N. benthamiana* leaves, a commonly used plant expression system. PGDH3, a stromal enzyme involved in maintaining chloroplast redox balance through NAD(H)‐dependent activity crucial for photosynthesis (Höhner et al., 2021; Krämer et al., 2024), served as a representative test protein.

**Figure 4:**
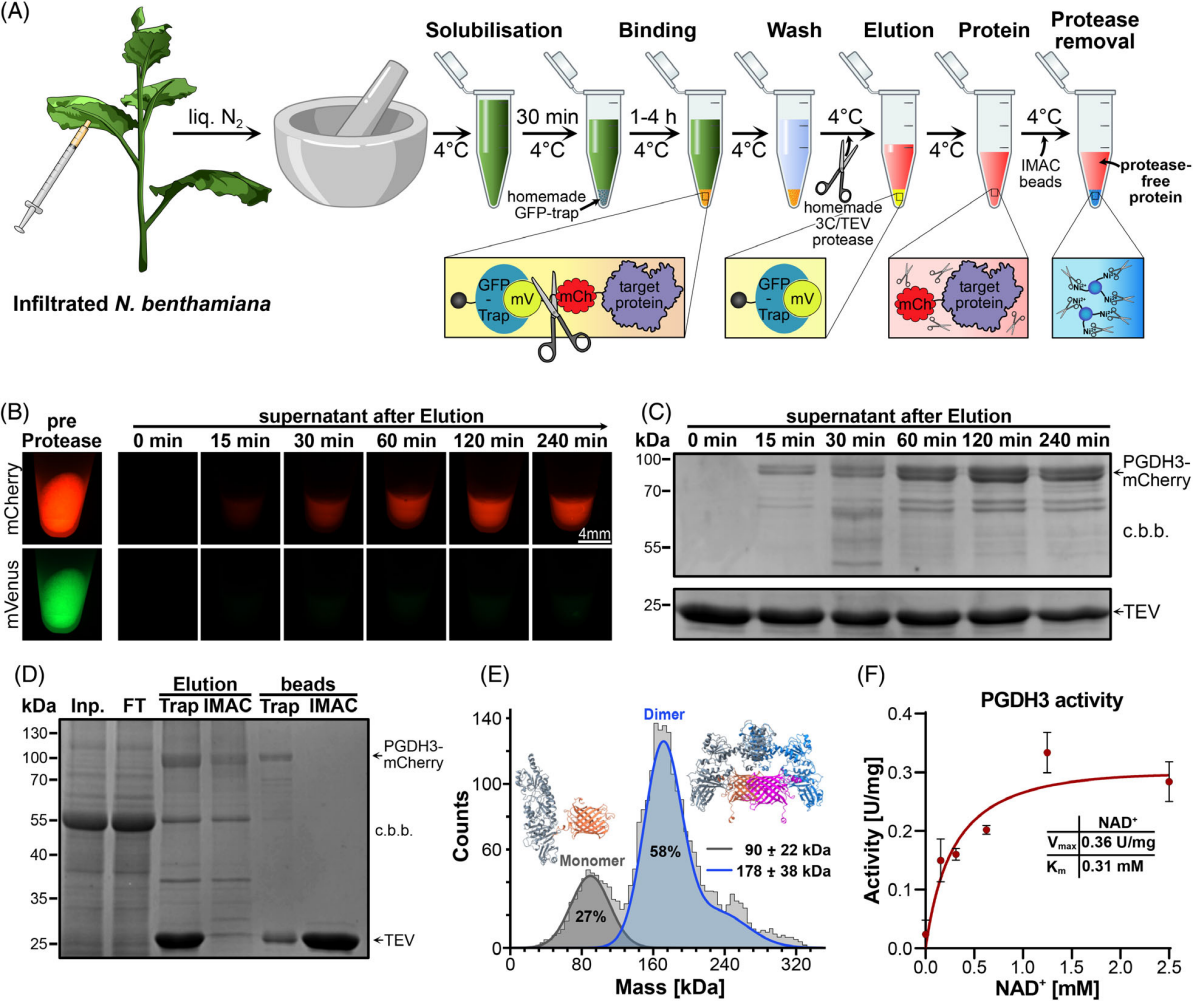
Double‐fluorescent tag purification and characterization of PGDH3‐mCherry. (A) Schematic representation of the ‘Catch & Release’ purification workflow using the double‐fluorescent tag. Transiently protein expressing *Nicotiana benthamiana* leaves were ground, solubilized, and incubated with GFP‐Trap resin to capture the fusion protein, followed by a series of wash steps. On‐resin digestion with a His‐tagged protease then releases the mCherry‐tagged target protein from the beads. The eluate, containing both the target protein and the protease, is subjected to immobilized metal affinity chromatography (IMAC). This step removes the His‐tagged protease, resulting in a final sample of pure, protease‐free mCherry‐tagged target protein. For information on volumes please refer to the Materials and Methods section. (B) Time‐resolved elution of PGDH3‐mCherry‐TEV‐mVenus. The left image depicts the fluorescence of PGDH3 bound to GFP resin before protease digestion. After enzymatic cleavage, eluted protein (free of resin) was collected at different time points and imaged under a fluorescence stereoscope. PGDH3‐mCherry‐TEV‐mVenus was transiently expressed utilizing the Catch & Release vectors including the UBQ10 promoter. (C) SDS‐PAGE analysis of elution fractions from (B), stained with Coomassie brilliant blue (c.b.b.). TEV protease served as a loading control. (D) SDS‐PAGE analysis stained with Coomassie brilliant blue (c.b.b.) illustrating PGDH3‐mCherry purification, including removal of His‐tagged TEV protease using Ni‐NTA affinity chromatography (IMAC). Lanes represent: Total protein lysate (Input, Inp.); unbound fraction after GFP‐Trap incubation (Flow‐Through, FT); protein eluted from GFP‐Trap after TEV cleavage (Elution Trap); final purified protein after IMAC step (Elution IMAC); GFP‐Trap resin post‐elution (beads Trap); IMAC resin post‐elution (beads IMAC). (E) Mass distribution histogram of purified PGDH3‐mCherry obtained using the isolation protocol. The histogram represents mean trajectory contrasts detected in a dynamic mass photometry analysis (*n* = 1 movie, 1 min), including trajectories of at least 151 ms in length (*n* = 2847 trajectories). Percentages correspond to the fraction of counts for each peak. Predicted 3D structures of both isoforms were generated using AlphaFold 3.0 (Abramson et al., 2024). (F) Michaelis‐Menten kinetics of purified PGDH3‐mCherry, assessing phosphoglycerate dehydrogenase function. Data are presented as mean ± SEM (*n* = 3).

Transient expression of PGDH3 fused to the mCherry‐TEV‐mVenus tag was achieved by agroinfiltration of *N. benthamiana* leaves. The protein purification strategy employed was analogous to that developed for stably transformed *A. thaliana* lines (Figure 3A), utilizing GFP‐Trap resin to capture the fusion protein via the mVenus moiety (Figure 4A). A distinct advantage of this dual‐tag system is the ability to monitor the elution process in real time. This is done by observing the fluorescence of the mCherry tag released into the supernatant. Upon incubation with TEV protease, the mVenus portion of the fusion protein remained bound to the GFP‐Trap resin, while only the PGDH3‐mCherry fragment was eluted (Figure 4A–C). Analysis of the elution kinetics using the TEV protease by SDS‐PAGE and tracking the mCherry fluorescence in the supernatant showed that the majority of the PGDH3‐mCherry protein was released within the first 2 h (Figure 4B,C). Tracking the elution kinetics can considerably shorten incubation times favorable for unstable proteins.

The use of HRV 3C protease significantly accelerated substrate cleavage, reducing the incubation time for less stable target proteins from ~120 min (TEV) down to ~30 min for PGDH3 (Figure S2E). This enhanced rate can be attributed to the higher activity and broader buffer compatibility of HRV 3C protease (Ullah et al., 2016).

A common challenge in protease‐assisted protein purification is the co‐elution of the protease itself. To address this, we implemented a subsequent purification step using reverse immobilized metal affinity chromatography (IMAC). Since the TEV protease possesses a His‐tag, incubation of the eluted PGDH3‐mCherry sample with Ni‐NTA resin effectively captured the protease, as shown by SDS‐PAGE analysis (Figure 4D). The purification of protease‐free PGDH3‐mCherry yielded ~100 μg protein g^−1^ ground plant material, that is, considerably higher amounts compared with the KEA1 isolation.

### One‐step purified PGDH3 via Catch & Release is of high quality

As aggregation can be a significant issue during protein purification, we assessed the dispersity and oligomeric state of protease‐free PGDH3‐mCherry purified via the ‘Catch & Release’ toolbox using mass photometry (Figure 4E). The results revealed two populations. The major species exhibited an average mass of 178 ± 38 kDa, while a smaller population (approximately one‐third of events) averaged at 90 ± 22 kDa. No major protein aggregates at higher molecular weights were detected. Considering the theoretical molecular weight of the PGDH3‐mCherry monomer of 87 kDa, these results strongly suggest that the purified protein exists predominantly as a dimer (~179 kDa), with a smaller fraction present as monomers (~90 kDa). This observation aligns with previous findings that human PGDH requires dimerization for its enzymatic activity (Xu et al., 2021), suggesting that the transient expression and purification protocol yielded fully functional, non‐aggregated, and correctly folded plant PGDH3. Both PGDH3 peaks accounted for 85% of the total signals, indicating high purity.

To further confirm the protein quality, we investigated the activity of purified PGDH3‐mCherry through an enzymatic assay. The protein exhibited NAD‐dependent dehydrogenase activity, consistent with its known function (Benstein et al., 2013). From Michaelis–Menten kinetics, we determined a *K*_m_ for NAD^+^ of 0.31 mM (at pH 8.0) and a *V*_max_ of 0.36 U mg^−1^. Notably, this *K*_m_ value aligns remarkably well with that reported for Arabidopsis PGDH3 purified from *E. coli* (0.24 mM, pH 8.1; Benstein et al., 2013), suggesting the protein purified from *N. benthamiana* shares similar kinetic properties. Ultimately, the measured activity provides clear evidence that the PGDH3‐mCherry protein, purified using the dual‐tag vector following transient expression in *N. benthamiana*, is enzymatically functional.

Taken together, these findings demonstrate that our novel mCherry‐TEV‐mVenus dual‐tag vector enables the rapid production and purification of substantial amounts of intact, pure, and biologically active plant proteins via transient expression, illustrated here with *A. thaliana* PGDH3 expressed in *N. benthamiana*. This approach significantly shortens the experimental timeline, requiring only about 1 week (~1‐day active work) compared with establishing stable *A. thaliana* lines.

### Comparison of Catch & Release toolbox with other plant purification workflows

To classify the performance of the ‘Catch & Release’ workflow, we compared it against four established purification protocols from plants, two each from *N. benthamiana* and *A. thaliana* (Figure S3D; Book et al., 2010; Sainsbury et al., 2016; Jeong et al., 2018; Islam et al., 2019). Except for Method 3, all other methods required substantially more handling time and procedural steps than the workflow presented here, which is particularly critical for unstable proteins or those prone to aggregation. Proteins could be isolated faster using Method 3. However, this method had a 20‐fold lower yield and used costly commercial components. While two methods (Method 1 and 4) achieved higher absolute yields, they required more time, and in part, relied on expensive commercial reagents. Purities were estimated by different methods, but all workflows reported comparable results, ranging from 85 to 95%. Notably, the ‘Catch & Release’ workflow was the only purification approach enabling *in vivo* localization and real‐time elution kinetics to be monitored with the same tag. In summary, the ‘Catch & Release’ workflow offers a unique combination of speed, high purity, high yield, cost‐effectiveness, and broad applicability.

### Considerations and limitations of the Catch & Release toolbox

While broadly applicable for most protein targets, using the Catch & Release toolbox requires previous considerations and has limitations. To access the full potential of the toolbox and its components, a minimum level of biochemical expertise and equipment is required. Although all proteins included in the toolbox (GFP‐clamp, TEV, and 3C) can be batch purified, we recommend further purification by size exclusion chromatography (SEC) on a fast protein liquid chromatography (FPLC) system to prevent contamination of the final sample.

The strategy relies on a GFP/YFP tag, which in rare cases may interfere with protein function. To minimize disruption of N‐terminal signal peptides and PTMs, vectors were intentionally designed with C‐terminal tags. Nevertheless, for some proteins, this tag placement may still affect correct targeting and/or function. Consequently, functional validation of tagged proteins through complementation experiments, or at a minimum, microscopic verification of correct localization is strongly recommended. In the future, we envision designing N‐terminally tagged vector variants. However, we also invite the community to modify Catch & Release plasmids as needed.

Furthermore, saturating the resin's binding capacity is recommended to maximize purity. This can be tested by analyzing the flow‐through for residual target protein. If no target protein is detected, the protein extract/GFP‐Trap ratio can be shifted toward more extract, combined with an increased incubation time.

Another aspect which requires attention is that some proteins tend to bind non‐specifically to the IMAC resin during the protease removal step, even without a poly‐His‐tag. Addition of up to 40 mM imidazole prior to resin addition can reduce this non‐specific binding. Alternatively, target protein and protease can be separated by SEC if there is a sufficient size difference. SEC requires specialized equipment, is more time‐consuming, and results in the dilution of the sample. Nevertheless, this step might result in higher protein purity and removal of soluble protein aggregates.

## CONCLUSIONS

Here, we developed and validated the ‘Catch & Release’ system, a flexible toolbox accelerating cloning, transgenic plant generation (via FAST), and protein purification from plants. Its modular design features efficient screening markers, diverse and precisely removable tags, including a novel dual‐fluorescence option. All modules are easily exchangeable to cater to individual needs. We demonstrated *in vivo* functionality of fusion proteins through successful KEA1 complementation in *Arabidopsis* loss‐of‐function mutants, confirming correct localization and function. Furthermore, by using DIY components, we established a robust, low‐cost, and fast one‐step purification workflow yielding tag‐free and functional proteins, demonstrated for both membrane (KEA1) and soluble (PGDH3) targets from stable or transient expression systems, respectively. Purification times are drastically shortened from days to hours, while yields (100 μg PGDH3 g^−1^ plant material) are sufficient for most downstream applications. Overall, Catch & Release provides an efficient and versatile platform for advancing plant protein biochemistry. We envision the workflow to be especially useful to rapidly prepare native plant protein isolates for structural analysis by Cryo‐EM, IP for interaction studies, or enzyme activity measurements.

## MATERIAL AND METHODS

### Plant growth and isolation of *A. thaliana* mutant lines

*Arabidopsis thaliana* (ecotype Col‐0) plants and *N. benthamiana* plants were grown on soil (Sungro Professional Growing Mix #1, Sun Gro Horticulture, Agawam, MA, USA) under long‐day conditions (16 h illumination/8 h dark) at 110 μmol photons m^−2^ sec^−1^, 22°C, 50% humidity. Rosettes of 3‐week‐old *A. thaliana* plants were used for all experiments if not stated differently. Homozygous genotypes were confirmed by PCR using primer combinations depicted in Table S1.

### Cloning

The *UBQ10* promoter in the pG20_Venus_Hyg vector (Pratt et al., 2020; Addgene ID: 159703) was first modified by site‐directed mutagenesis PCR using primers pGII_UBQ_1 and pGII_UBQ_2 to remove a BbsI restriction site. Subsequently, a synthetic DNA fragment (Geneart, Thermo, Waltham, MA, USA) containing a constitutively expressing RFP cassette flanked by two BbsI Golden Gate sites and a TEV protease site with a downstream NcoI site was inserted via Gibson Assembly into the BamHI/XmaI‐linearized pG20_updated‐UBQ10_Venus_Hyg vector.

To introduce various epitope tags, the pG20_updated‐UBQ10_RFP‐GoldenGate_TEV_Venus_Hyg vector was linearized with NcoI and SacI (New England Biolabs, Frankfurt, Germany). Flag, Strep, MYC, and HA tags were incorporated by annealing complementary oligonucleotides (listed in Table S1) and ligating them into the linearized vector. For mCherry insertion, the NcoI site was first removed by amplifying two fragments from the pG20_mCherry_Hyg vector (Pratt et al., 2020; Addgene ID: 159701) using primers pGII_mCherry_1 to pGII_mCherry_4, followed by Gibson Assembly, employing the same strategy used for the epitope tags.

The FAST‐Red cassette was amplified from synthetic DNA (Geneart, Thermo) using primers pGII_FAST_1 and pGII_FAST_2, while the pG20_Venus_Hyg vector was amplified with primers pGII_FAST_3 and pGII_FAST_4. Gibson Assembly resulted in the pG20_FAST‐Red (FR) Venus vector, which was subsequently digested with HindIII (upstream of the UBQ10 promoter) and EcoRI (downstream of the HSP18.2 terminator) to introduce the updated expression cassette described above. This construct incorporated the UBQ10 promoter, RFP cassette, TEV protease cleavage site, selected tags, and the HSP18.2 terminator. The final plasmids were designated as pG20_TEV_tag_FR.

For the FAST‐Green cassette, the pG20_TEV_mVenus_FR vector was amplified using primers pGII_FRed_1 and pGII_FRed_2, while eGFP was amplified using primers pGII_FGreen_1 and pGII_FGreen_2. The two fragments were assembled via Gibson cloning, yielding the pG20_TEV_mVenus_FG vector. Subcloning of all the tag variants followed the same strategy as for the FAST‐Red vector, utilizing HindIII and EcoRI restriction sites.

The TEV protease cleavage site in the pG20_TEV_mVenus_FR vector was replaced by digesting the vector with XmaI (New England Biolabs, Frankfurt, Germany) and NcoI, followed by ligation of annealed primers pGII_3C_1 and pGII_3C_1.

### Cloning and efficiency comparison

To compare the efficiencies of different cloning methods, the *PGDH3* (AT3G19480) coding sequence (Lopez et al., 2022) was cloned into the pG20_TEV‐mVenus_FR vector. The *PGDH3* insert was amplified by PCR using method‐specific primers (Table S1). Each cloning reaction was performed in triplicate.

The restriction–ligation was performed by digesting both the pG20_TEV‐mVenus_FR vector and the PCR‐amplified *PGDH3* insert with BamHI and XmaI (New England Biolabs, Frankfurt, Germany). Subsequently, 50 ng of the digested vector was ligated with 34 ng of the digested insert using T4 DNA Ligase (NEB) in a 20 μl reaction volume for 15 min at room temperature (RT).

For Gibson Assembly, the pG20_TEV‐mVenus_FR vector was linearized with BamHI and XmaI. The assembly reaction contained 50 ng of the linearized vector, 34 ng of the *PGDH3* insert (amplified with Gibson‐specific primers), and 10 μl of a homemade 2× Gibson master mix (200 mM Tris–HCl pH 7.5, 20 mM MgCl_2_, 0.4 mM dNTPs, 20 mM DTT, 2 mM NAD, 8 U ml^−1^ T5 exonuclease [NEB], 50 U ml^−1^ Q5 polymerase [NEB], 32 U ml^−1^ Taq ligase [NEB], 10% [w/v] PEG 8000). The reaction was adjusted to a final volume of 20 μl with nuclease‐free water and incubated for 1 h at 50°C.

Golden Gate assembly utilized a one‐pot reaction where 50 ng of the circular pG20_TEV‐mVenus_FR plasmid was mixed with the *PGDH3* insert amplified with Golden Gate‐specific primers, T4 DNA Ligase (NEB), and the BbsI restriction enzyme (NEB). The reaction was subjected to 25 cycles of digestion (37°C for 5 min) and ligation (16°C for 5 min), followed by a final heat inactivation step at 80°C for 10 min.

Following each cloning reaction, 4 μl of the mixture was transformed into chemically competent *Escherichia coli* DH5α cells. 150 μl of cell suspension from each transformation was plated on selective agar plates. To assess cloning efficiency, colony PCR was performed on 15 randomly selected white colonies from each plate using primers listed in Table S1.

### Assessment of expression host for experimental protein structure of Arabidopsis proteins

A search of the PDB (https://www.rcsb.org/) was performed in September 2025 to identify experimental and integrative structures with *A. thaliana* as the source organism. The resulting dataset, including PDB ID, source organism, and expression host, was downloaded. Entries lacking PDB IDs or information on the expression host were excluded. After filtering, 2412 PDB entries remained, comprising 14 entries (0.7%) with Arabidopsis and 1975 entries (82%) with *E. coli* listed as expression hosts.

### Stable transformation of *A. thaliana*

*KEA1* (AT1G01790) genomic DNA (Bölter et al., 2020) and *PGDH3* (AT3G19480) cDNA (Lopez et al., 2022) were amplified without their stop codon by PCR using primers listed in Table S1 and cloned in the pG20_TEV‐mVenus_FR, pG20_TEV‐mVenus_FG, pG20_3C‐mVenus_FR, pG20_TEV‐mCherry_FR, pG20_TEV‐Flag_FR, pG20_TEV‐Strep_FR, pG20_mCherry_TEV‐mVenus_FR, pG20_TEV‐MYC_FR, and pG20_TEV‐HA_FR Catch & Release vectors (listed in Table 1) by classical restriction cloning using BamHI/XmaI restriction sites. *kea1‐1kea2‐1* mutant plants were previously described by Kunz et al. (2014). *Arabidopsis thaliana kea1‐1kea2‐1* plants were transformed with the vectors containing fluorescently tagged *KEA1* (listed in Table S2) using the floral dip method (Clough & Bent, 1998) with the *Agrobacterium tumefaciens* GV3101 strain carrying the additional helper plasmid pSOUP (Hellens et al., 2000). Transgenic seeds were screened for GFP or RFP fluorescence using the Leica M165 FC Fluorescent Stereoscope (Leica, Wetzlar, Germany). Microscopy was carried out in the T_1_ generation. Immunoblotting was performed in fluorescence‐selected homozygous F_3_ plants.

To assess the efficiency of transgenic line selection, genotyping was performed on approximately >79 plants transformed with Catch & Release vectors containing either the Fast‐Red or HYGROMYCIN resistance cassettes. Vector‐specific primers (listed in Table S1) were utilized for PCR‐based genotyping. To preclude false‐negatives, the integrity of the isolated genomic DNA was confirmed through a separate, confirmatory PCR analysis.

### Transient transformation of *N. benthamiana*

For overexpression and IP studies, *N. benthamiana* leaves were infiltrated with *A. tumefaciens* strains carrying *KEA1* and *PGDH3* containing vectors (listed in Table S2) as well as co‐injected with the 19k vector according to Waadt et al. (2014). Infiltrated leaves were harvested from plants after 6 days of greenhouse culturing.

### Protoplast isolation and confocal microscopy for protein localization

Protoplasts were prepared according to the ‘Tape‐Arabidopsis‐Sandwich’ method (Wu et al., 2009). Imaging was carried out utilizing a Leica Stellaris 5 Confocal Laser Scanning Microscope (Leica, Wetzlar, Germany), featuring a supercontinuum White Light Laser (WLL) and a 405 nm diode. Emission signals were detected using a Power Hybrid HyDS detector. For protein localization, recombinant mVenus (YFP)‐tagged proteins were excited at 514 nm, with emission recorded between 520 and 580 nm. Similarly, mCherry (RFP)‐tagged proteins were excited at 585 nm, and emission was detected within the 615–670 nm range. Chlorophyll autofluorescence (chl a) was excited at 405 nm, with emission captured between 623 and 813 nm. Protoplast imaging was performed via Z‐stack acquisition, optionally enhanced using the LIGHTNING module, and processed in LAS X software to generate maximum intensity projections.

### Photosynthetic measurements

Photosynthetic performance was evaluated using a Walz Imaging PAM system (Walz GmbH, Effeltrich, Germany) following the methodology outlined by Kunz et al. (2009). Prior to measurements, plants underwent a 30‐min dark acclimation period. A standard induction curve was applied at an irradiance of 110 μmol photons m^−2^ sec^−1^ for a duration of 300 sec, with measurements acquired at 20‐sec intervals. False‐color image analysis and data export were conducted using the Walz ImagingWinGigE software.

### Plant protein purification

Three‐week‐old *A. thaliana* plants or infiltrated *N. benthamiana* leaves were harvested and ground to a fine powder in liquid nitrogen. Protein extraction was carried out by gently resuspending the plant powder in an extraction buffer (50 mM Tris–HCl pH 7.5, 150 mM NaCl, 2.5 mM EDTA, 10% [v/v] glycerol, 0.5% [w/v] Triton X‐100 [KEA1 isolations]/NP‐40 [PGHD3 isolations], 5 mM DTT, 1× protease inhibitors) at a ratio of 1.5 ml of buffer per gram of powder and incubated on a rotation shaker at 4°C for 30 min. Following centrifugation (25 000 ***g***, 20 min, 4°C), the supernatant was filtered and incubated with homemade GFP‐Trap resin for 4 h at 4°C. The quantity of resin was adjusted based on the target protein's expression level: 20–50 μl of resin (bed volume) per 1.5 ml of protein extract for low to medium expression, and up to 100 μl for highly expressed proteins.

After the binding step, the resin was washed three times with wash buffer (50 mM Tris–HCl pH 7.5, 150 mM NaCl, 2.5 mM EDTA, 10% [v/v] glycerol, 0.5% [w/v] Triton X‐100 or NP‐40). Dependent on the final application, the stringency of the wash steps can be adjusted: For the isolation of a single protein, a high stringency can be used, that is, more than three washing steps, high wash buffer volumes (1.5 ml per wash), and increased detergence and/or salt concentrations. If protein interaction partners need to be preserved, 2–3 wash steps, low volumes (500 μl), and low detergent and salt concentrations are recommended.

Following the final wash, resin‐bound protein samples were prepared by resuspending the resin in 200 μl wash buffer. An aliquot of 40 μl representing the ‘Trap’ sample of this mixture was transferred to a new tube, centrifuged, and the supernatant removed.

For protein elution via on‐column protease digestion, the remaining resin was pelleted and resuspended in 200 μl of elution buffer (50 mM Tris–HCl pH 7.5, 150 mM NaCl, 4% [v/v] glycerol, 0.5% [w/v] Triton X‐100 [only for KEA1 isolation]) containing 15 μM TEV or 3C protease, and digestion was monitored over 4 h. The volume of elution buffer was adjusted to achieve the desired final protein concentration. For high concentration elutions, as was the case for KEA1, a minimum of a 1:2 ratio (e.g., 20 μl of resin bed volume to 40 μl of elution buffer) is recommended. However, for the elution of PGDH3, a ratio of 1:5 was employed, using 100 μl of elution buffer for every 20 μl of resin bed volume. Cleaved protein‐containing supernatants were then flash‐frozen in liquid nitrogen and stored at −80°C for downstream applications. After elution, the GFP‐Trap beads were washed three times with 1 ml elution buffer and prepared for SDS‐PAGE (as described below).

To remove the His‐tagged proteases, immobilized metal affinity chromatography (IMAC) was employed. Ni‐NTA Agarose beads (Machery‐Nagel, Düren, Germany) were added to a new Eppendorf tube, with the quantity determined according to the manufacturer's instructions. The Ni‐NTA beads were washed twice with 500 μl elution buffer. Imidazole was added to the protein‐containing supernatant to a final concentration of up to 40 mM. Subsequently, the solution was added to the equilibrated IMAC resin and incubated for 30 min on ice with constant mixing. Following centrifugation (1000 ***g***, 2 min, 4°C), the supernatant, containing the protease‐free protein, was transferred to a new tube, flash‐frozen in liquid nitrogen, and stored at −80°C. After protease removal, the IMAC beads were washed three times with 1 ml elution buffer and prepared for SDS‐PAGE (as described below).

All volumes can be proportionally scaled to the experimental needs.

For immunoblotting and SDS‐PAGE, samples (including resin‐bound fractions) were mixed with SDS loading buffer and heated at 80°C for 10 min. Samples were either stored at −20°C or used immediately.

### Mass photometry

Mass photometry measurements of PGDH3‐mCh were performed using a TwoMP instrument (Refeyn Ltd, Oxford, UK). Protein stocks were diluted in phosphate‐buffered saline (PBS; 137 mM NaCl, 2.7 KCl, 8.1 mM Na_2_HPO_4_, 1.5 mM KH_2_PO_4_) prior to analysis. Sample well cassettes (Ref: RD501078; Refeyn Ltd, Oxford, UK) were mounted on clean microscope coverslips (Ref: MP‐CON‐41001; Refeyn Ltd, Oxford, UK). For each measurement, a clean well was pre‐filled with 15 μl of PBS for autofocus stabilization. Subsequently, 5 μl of diluted protein solution was added to achieve a final protein concentration of 5 nM. Movies (60 sec duration) were recorded using standard instrument settings. Data were acquired using AcquireMP software and analyzed with DiscoverMP software (Refeyn Ltd, Oxford, UK). Each sample was measured at least three times (*n* ≥ 3). Instrument calibration was performed using a MassFerence P1 Calibrant (Refeyn Ltd, Oxford, UK). Protein structures were simulated using AlphaFold 3.0 (Abramson et al., 2024).

### Enzymatic activity assay

PGDH3 activity was assessed in a reaction buffer containing a final concentration of 100 mM Tris–HCl (pH 8.0), 5 mM hydrazine sulfate, 5 mM MgCl_2_, and 0–2.5 mM NAD^+^ (Höhner et al., 2021). Prior to the reaction, the protein sample was incubated separately at RT for 10 min with 20 mM DTT. Each of the three replicates consisted of 200 μl total volume with a final protein concentration of 2.5 ng μl^−1^. The reaction was initiated by adding 3‐phosphoglyceric acid to a final concentration of 10 mM. Absorbance change was recorded at 340 nm using a Spark Multimode Microplate Reader (Tecan, Männedorf, Switzerland). Using the GraphPad Prism Software (v. 10.4.2), absorbance values were fitted according to Michaelis Menten.

### Immunoblotting

Immunoblotting was performed on total leaf tissue from homozygous *A. thaliana* lines or infiltrated *N. benthamiana* leaves (Völkner et al., 2021, 2024). Equal amounts of leaf tissue were ground and extracted in solubilization buffer (50 mM Tris–HCl pH 7.5, 150 mM NaCl, 2 mM EDTA, 5 mM DTT, 10% [v/v] glycerol, 0.5% [w/v] Triton X‐100, plant protease inhibitor mix) for 30 min at 4°C. Samples were either incubated with the 3C or TEV protease for 1 h before mixing with SDS loading dye and boiling at 95°C for 5 min or directly mixed with SDS loading dye and boiled. Proteins were separated on 8–15% (w/v) SDS‐PAGE gels based on molecular weight and transferred to a PVDF membrane using a wet‐blotting system. Immunodetection was performed overnight at 4°C using primary antibodies (listed in Table S3) followed by chemiluminescent detection with HRP‐conjugated secondary antibodies.

### Protease purification

Plasmids encoding TEV or HRV‐3C protease (listed in Table S2) were transformed into *Escherichia coli* BL21 (DE3) and cultured in Terrific Broth (TB) medium supplemented with 50 μg ml^−1^ kanamycin at 37°C until the optical density at 600 nm (OD_600_) reached 0.6. Protein expression was induced with 0.5 mM isopropyl β‐D‐1‐thiogalactopyranoside (IPTG), followed by incubation at 18°C for 16 h. Cells were harvested by centrifugation (3500 ***g***, 15 min at 25°C), resuspended in buffer A (50 mM Tris–HCl, pH 8.0, 300 mM NaCl, 5% [v/v] glycerol, 2 mM DTT, 20 mM imidazole), flash‐frozen in liquid nitrogen, thawed on ice, and lysed using a tissue lyser.

Cell debris was removed by centrifugation (45 000 ***g***, 30 min at 4°C), and Poly‐Histidine‐tagged proteases were purified by IMAC using a HisTrap HP (Cytiva Life Science, Wilmington, DE, USA) column on an ÄKTA PURE FPLC (Cytiva Life Science, Wilmington, DE, USA) system. After washing with buffer A, proteins were eluted using buffer A supplemented with 300 mM imidazole. The buffer was subsequently exchanged to buffer B (50 mM Tris–HCl, pH 8.0, 150 mM NaCl, 5% [v/v] glycerol, 1 mM DTT) using a HiPrep 26/10 Desalting column (Cytiva Life Science, Wilmington, DE, USA).

Further purification was performed via SEC using a HiLoad 16/16 Superdex 75 or 200 pg column (Cytiva Life Science, Wilmington, DE, USA) equilibrated in SEC buffer (50 mM Tris–HCl, pH 8.0, 150 mM NaCl, 10% [v/v] glycerol, 1 mM DTT). Fractions were analyzed by SDS‐PAGE, pooled, concentrated to 3 mg ml^−1^, aliquoted, flash‐frozen in liquid N_2_, and stored at −80°C.

### Expression and purification of the GFP‐clamp (GFPc)

The homemade GFP‐Trap is based on a double DARPin (GFP‐clamp or GFPc) engineered for high‐affinity GFP binding (Hansen et al., 2017). The construct was obtained from the Plückthun Laboratory (University of Zurich, Zurich, Switzerland). The GFPc was subcloned into a pET‐28a vector, fusing it to an N‐terminal and TEV‐cleavable His10‐Thioredoxin (HSTrx) tag followed by a triple Lysine (KKK) sequence (Data S1 for the full amino acid sequence of fusion protein; full‐length: 47.8 kDa; cleaved: 32.2 kDa). The triple Lysine sequence ensures directional amine coupling of GFPc (see ‘Preparation of GFP‐Trap Affinity Resin’), which does not possess any other surface‐exposed primary amines, that is, Lysine residues (Hansen et al., 2017).

*Escherichia coli* Rosetta 2 (DE3) competent cells were transformed with the subcloned GFPc plasmid and cultured in TB medium supplemented with 50 μg ml^−1^ kanamycin and 34 μg ml^−1^ chloramphenicol at 37°C until an OD_600_ of 0.6 was reached. Protein expression was induced with 1 mM IPTG, followed by incubation at 20°C for 16 h. Cells were harvested by centrifugation (3000 ***g***, 10 min at 25°C), resuspended in buffer A (50 mM Tris–HCl, pH 8.0, 300 mM NaCl, 5% [v/v] glycerol, 2 mM ß‐mercaptoethanol, 20 mM imidazole), flash‐frozen in liquid nitrogen, thawed on ice, and lysed using a tissue lyser. The lysate was clarified by centrifugation at 30 000 ***g*** for 30 min at 4°C. The supernatant was collected and filtered through a 0.45 μm syringe filter.

Protein purification was performed at 4°C using an ÄKTA FPLC system. Filtered supernatant was applied to a HisTrap HP (Cytiva Life Science, Wilmington, DE, USA) column equilibrated in buffer A. The column was washed sequentially with 5–10 column volumes (CV) of buffer A, 5 CV of buffer C (50 mM Tris–HCl pH 8.0, 1 M NaCl, 5% (v/v) glycerol, 20 mM imidazole), and finally 5 CV of buffer A before elution with buffer B (50 mM Tris–HCl pH 8.0, 300 mM NaCl, 5% [v/v] glycerol, 300 mM imidazole). Pooled fractions were buffer‐exchanged into PBS (137 mM NaCl, 2.7 KCl, 8.1 mM Na_2_HPO_4_, 1.5 mM KH_2_PO_4_, pH 7.4) using a HiPrep 26/10 Desalting column. The HSTrx tag was cleaved overnight at 4°C using homemade histidine‐tagged TEV protease (1:100 w/w ratio). The cleaved tag and TEV protease were removed by passing the reaction over a second HisTrap HP column equilibrated in PBS; the KKK‐GFPc was collected in the flow‐through. Final purification was achieved by SEC on a HiLoad 16/600 Superdex 200 pg column equilibrated in PBS. Monomeric KKK‐GFPc fractions (eluting at ~85 ml) were pooled and concentrated. Protein concentration was determined by A280 (*ε*_molar_ = 29.000 M^−1^ cm^−1^). Purified protein was flash‐frozen and stored at −80°C.

### Preparation of GFP‐Trap affinity resin

Purified, tag‐cleaved KKK‐GFPc was covalently immobilized onto NHS‐activated Sepharose 4 Fast Flow resin (Cytiva Life Sciences, Wilmington, DE, USA, Cat# 17090601) via amine coupling, targeting the N‐terminal KKK sequence. All steps were performed at RT. The vendor‐supplied resin slurry was transferred to a conical tube, pelleted by centrifugation (1500 ***g***, 5–10 min), and the storage solution was discarded. The resin was washed two times in 50 ml 1 mM HCl (centrifugation at 1500 ***g***, 5–10 min) then activated by a 10‐min incubation in 1 mM HCl on a rotating shaker. Subsequently, the resin was washed twice with PBS (137 mM NaCl, 2.7 mM KCl, 8.1 mM Na_2_HPO_4_, 1.5 mM KH_2_PO_4_, pH 7.4).

Following the final PBS wash and pelleting, the settled resin bed volume was estimated. Purified KKK‐GFPc diluted in PBS (0.8 mg GFPc ml^−1^ resin bed volume) was added to the activated resin in a final volume of 50 ml. The mixture was incubated for 2 h at RT with rotation to facilitate coupling.

After coupling, the resin was pelleted, and the supernatant was removed. No residual GFP‐clamp protein could be detected in the flow‐through after coupling. To block any remaining active NHS esters, the resin was resuspended in 100 mM Tris–HCl pH 8.5 containing 150 mM NaCl in a total volume of 50 ml and incubated for 1 h at RT with rotation. The coupled and blocked resin was subsequently washed by sequential resuspension (to approx. 50 ml) and pelleting: twice with PBS containing 1 M NaCl, and once with PBS containing 20% (v/v) ethanol. Finally, the pelleted resin was resuspended in a volume of PBS containing 20% (v/v) ethanol equal to the settled resin bed volume, creating a 50% (v/v) slurry for storage at 4°C. The resulting GFP‐Trap is stable for at least 2 years.

The long‐term binding stability of a homemade GFP‐Trap resin was assessed using three different batches: freshly prepared, 2‐year‐old, and 5‐year‐old resin. Each batch had a theoretical binding capacity of 0.8 mg ml^−1^.

A total of 30 μl of each GFP‐Trap resin batch was washed twice with elution buffer (50 mM Tris–HCl pH 7.5, 150 mM NaCl, 4% v/v glycerol). Following the wash, the resin was incubated with 48 μg of purified GFP protein for 2 h on ice. This step was performed three times for each batch.

After incubation, the resin was pelleted by centrifugation for 1 min at 1000 ***g***. The supernatant, containing unbound GFP, was collected and quantified using a NanoDrop One spectrophotometer (Thermo Fisher Scientific, Waltham, MA, USA) at a wavelength of 280 nm. Raw absorption values were adjusted with the molar extinction coefficient. The total amount of GFP bound to the resin was then determined by subtracting the amount of unbound GFP from the initial input of 48 μg.

### Key materials

**Table:** 

Resource	Source
Catch & Release vector set	Addgene IDs: 240650‐240679; European Plasmid Repository IDs: 983‐1012
TEV protease	Addgene ID: 171782
3C protease	Addgene ID: 162795
GFP‐clamp	(Hansen et al., 2017), see Data S1
Resin for GFP‐Trap	Cytiva Life Science, Prod. #: 17090601

## ACCESSION NUMBERS

The following genetic loci were important to this project: KEA1 (AT1G01790), KEA2 (AT4G00630), PGDH3 (AT3G19480). *Arabidopsis thaliana* (*A. thaliana*) wild‐type and T‐DNA insertion mutant plants employed in this study were all from the Columbia 0 (Col‐0) accession. The homozygous T‐DNA insertion mutant *kea1‐1kea2‐1* (SAIL_586_D02, SALK_045324) was published previously (Kunz et al., 2014) and can be obtained from the stock centers under the following IDs: ABRC: CS72318 or NASC: N72318.

## AUTHOR CONTRIBUTIONS

BB and HHK designed the project. SS performed most experiments, analyzed data, and prepared figures. SS and BB designed figures. BB, SS, and CE cloned constructs. CE and SS established transgenic plants. SM carried out CLSM, designed the Catch & Release logo, and created the figure icons for tobacco and Arabidopsis. EW performed PGDH assays. TW helped with mass photometry and carried out KEA1 IP. SS, BB, and HHK wrote the manuscript. All authors assisted in editing the manuscript.

## CONFLICT OF INTEREST

All author(s) declare no competing interests.

## Supporting information


**Figure S1.** Detailed map of cloning sites and cloning strategy guide for the Catch & Release system.

**Figure S2.** Purification and activity assessment of recombinant TEV and HRV 3C proteases.

**Figure S3.** Preparation, stability, and cost‐effectiveness of the homemade GFP‐Trap.

**Table S1.** List of primers used in the present study.

**Table S2.** List of vectors used in this study.

**Table S3.** Antibodies, bacterial strains, chemicals, software, and plant lines used in this study.

**Table S4.** PDB IDs from Arabidopsis protein structures including expression host information.

**Data S1.** Amino acid sequence of GFP‐clamp including N‐terminal tags.

## Data Availability

All data supporting the findings of this study are available within the paper and within the supplementary data published online. Vectors used in this study are listed in Table 1 and Table S2. All vectors and can be obtained from Addgene and the vectors listed in Table 1 are additionally available at the European Plasmid Repository (EPR).
