# Supplementary material for: Catch & Release—rapid cost‐effective protein purification from plants using a DIY GFP‐Trap‐protease approach
Source: Plant J. 2025 Nov 12;124(3):e70544. doi: 10.1111/tpj.70544 (PMC12611452; doi:10.1111/tpj.70544)
Supplement: Supplementary file 1 — Figure S1. Detailed map of cloning sites and cloning strategy guide for the Catch & Release system. [file TPJ-124-0-s005.pdf]

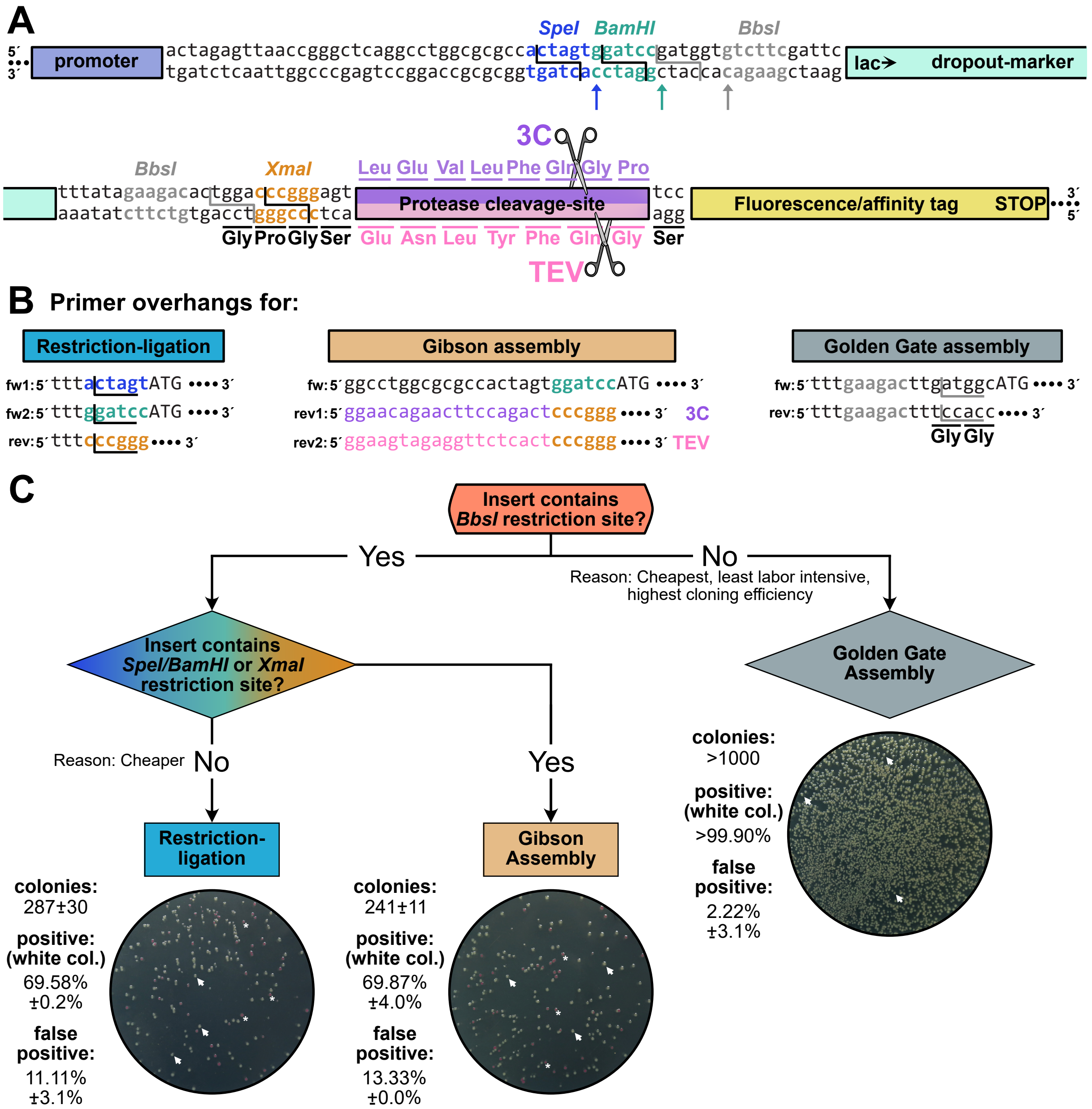

Figure S1. Detailed map of cloning sites and cloning strategy guide for the Catch & Release system.

(**A**) Detailed map of the multiple cloning site within the Catch & Release vectors. Restriction sites are shown for conventional cloning (SpeI, blue; BamHI, cyan; XmaI, orange), Gibson assembly (BamHI, XmaI), and Golden Gate assembly (BbsI, grey). Coloured arrows indicate the required position of the start codon (ATG) to ensure an in-frame fusion with the tag. The encoded amino acid sequences of the linkers, the recognition and cleavage sites for 3C (purple) and TEV (pink) proteases are provided. (**B**) Recommended primer overhangs for the indicated cloning methods. Forward primers (fw) are designed to include the start codon. The reverse primer (rev) design for Gibson assembly depends on the vector's specific protease cleavage site (3C or TEV). The reverse primer for Golden Gate assembly adds a terminal glycine residue. The primer overhangs are also listed in tab. S1 (**C**) A flowchart to guide the selection of an appropriate cloning strategy based on insert characteristics. The chart includes representative colony counts (total red and white colonies) and the positive cloning rate, defined as the ratio of positive clones (white colonies, indicated by arrows) to total colonies. Red colonies (asterisks) represent negative clones lacking an insert. False positive colonies were verified by colony PCR. Data are presented as mean ± SEM (n = 3).
