# Supplementary material for: Catch & Release—rapid cost‐effective protein purification from plants using a DIY GFP‐Trap‐protease approach
Source: Plant J. 2025 Nov 12;124(3):e70544. doi: 10.1111/tpj.70544 (PMC12611452; doi:10.1111/tpj.70544)
Supplement: Supplementary file 2 — Figure S2. Purification and activity assessment of recombinant TEV and HRV 3C proteases. [file TPJ-124-0-s002.pdf]

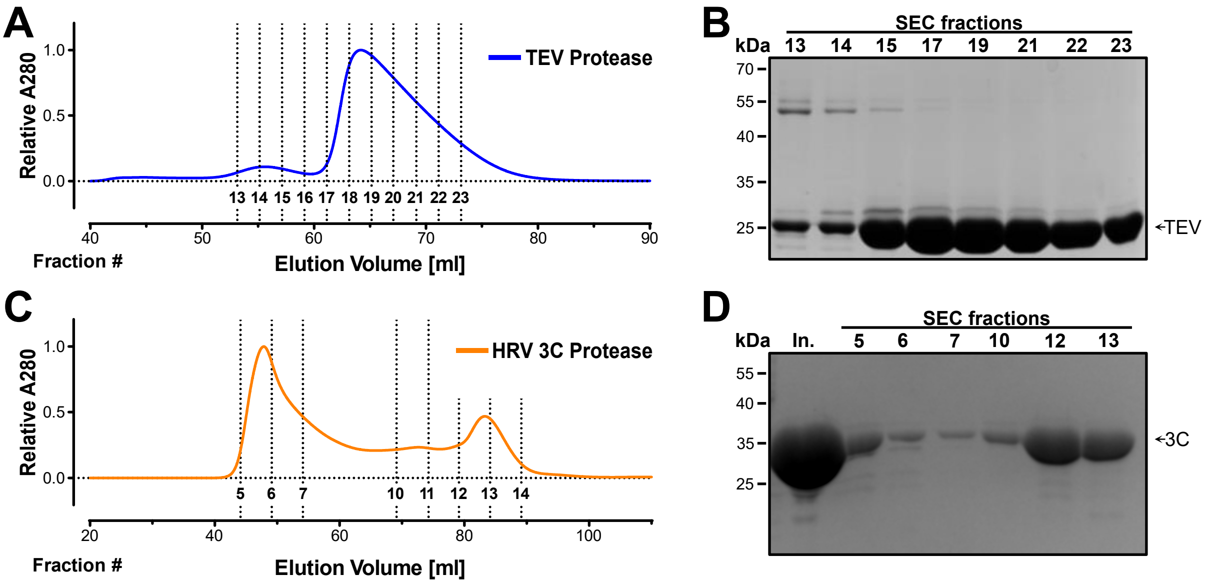

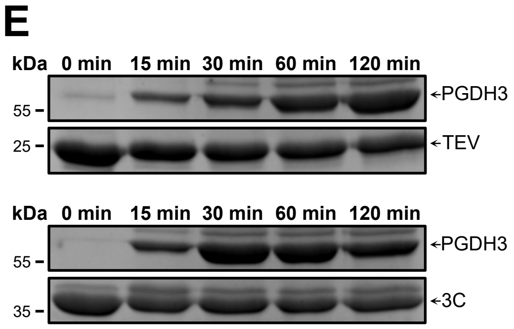

F

	Protease	Workforce	Material	Total cost
**homemade**	TEV / 3C protease overexpression plasmid **~80€/plasmid**	3 days of work **~500-1000€ / 240 mg**	batch (Ni-NTA) purification/ FPLC with IMAC and SEC column	**~2.5 - 4.5€ / mg** ~580-1080€ / 240 mg protease
**commercial 1 (TEV)**	TEV protease (Sigma-Aldrich)	/	/	**~376€ / mg** ~753€ / 10.000U
**commercial 2 (TEV)**	AcTEV™-protease	/	/	**~1020€ / mg** ~2040€ / 10.000U
**commercial 1 (3C)**	HRV-3C protease (Sigma-Aldrich)	/	/	**~354€ / mg**
**commercial 2 (3C)**	Pierce™ HRV 3C protease	/	/	**~682€ / mg** ~3410€ / 10.000U

Figure S2. Purification and activity assessment of recombinant TEV and HRV 3C proteases.

(**A**) Size exclusion chromatography (SEC) chromatographs of recombinantly expressed tobacco etch virus (TEV) protease purified using a HiLoad 16/60 Superdex 75 pg column. Dashed lines indicate collected fractions, with fraction numbers shown below. (**B**) SDS-PAGE analysis of TEV protease gel filtration fractions stained with Coomassie blue. Fractions 19–23 were pooled, concentrated, and used in subsequent experiments. (**C**) SEC elution profile of recombinantly expressed human rhinovirus (HRV) 3C protease purified using a HiLoad 16/60 Superdex 200 pg column. Dashed lines indicate collected fractions, with fraction numbers shown below. (**D**) SDS-PAGE analysis of HRV 3C protease gel filtration fractions stained with Coomassie blue. Fractions 12–13 were pooled for further analyses. (**E**) Coomassie stained SDS-PAGE comparing cleavage efficiency between homemade TEV and HRV 3C proteases over a 120-minute incubation with PGDH3-mVenus bound to homemade GFP-trap resin. Both proteases were used at a final concentration of 12 µM. (**F**) Cost comparison between homemade and commercial proteases. The cost per milligram of homemade TEV and 3C proteases is compared to commercially available equivalents: Sigma-Aldrich TEV protease (ProductID: T4455-10KU), ThermoFisher Scientific AcTEV™-protease (ProductID: 12575023), Sigma-Aldrich HRV-3C protease (ProductID: SAE0045-1MG) and ThermoFisher Scientific Pierce™ HRV 3C protease (ProductID: 88947). For AcTEV™ protease, we assumed that 10.000 U corresponds to 2 mg of protease, based on its comparable cleavage activity to the Sigma-Aldrich TEV protease, where 10.000 U is defined as 2 mg of protease. The production of 240 mg homemade protease can be completed in three days, requiring access to an FPLC system equipped with a size exclusion and IMAC column. Alternatively, batch purification using Ni-NTA beads is possible. Prices are subject to regional variation and might change.
