# Supplementary material for: Catch & Release—rapid cost‐effective protein purification from plants using a DIY GFP‐Trap‐protease approach
Source: Plant J. 2025 Nov 12;124(3):e70544. doi: 10.1111/tpj.70544 (PMC12611452; doi:10.1111/tpj.70544)
Supplement: Supplementary file 3 — Figure S3. Preparation, stability, and cost‐effectiveness of the homemade GFP‐Trap. [file TPJ-124-0-s001.pdf]

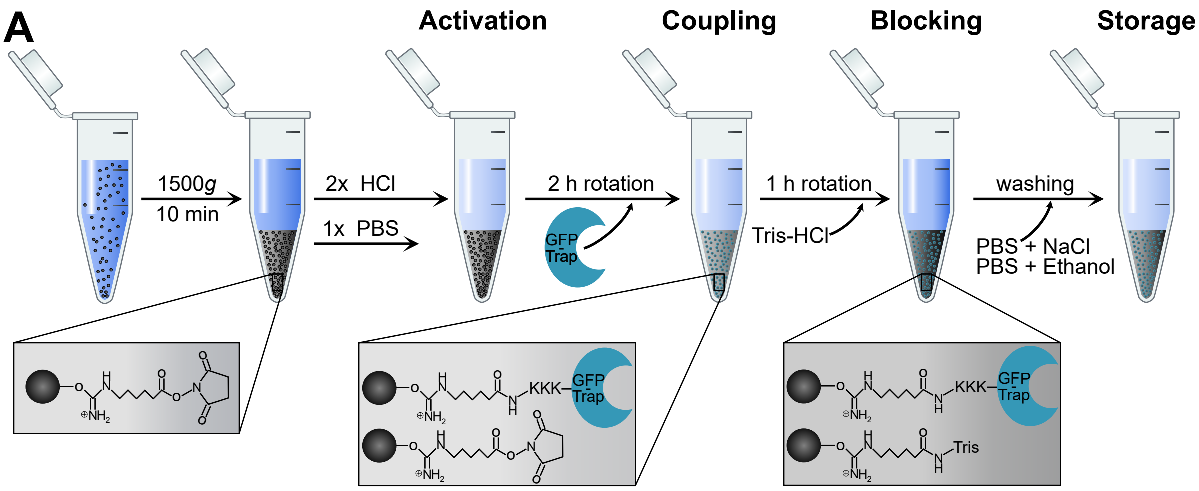


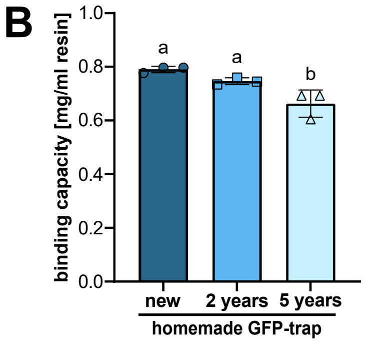


**C**

| | Resin | Workforce | Material | Total cost |
|---|---|---|---|---|
| **homemade** | NHS-Activated Sepharose 4 Fast Flow resin **~290€ / 50 ml slurry** | 3 days of work **~500-1000€ / 50 ml slurry** | batch (Ni-NTA) purification/ FPLC with IMAC and SEC column | **~15.8-25.8€ /ml slurry** ~790-1290€ / 50 ml slurry |
| **commercial 1** | ChromoTek GFP-Trap® Agarose | | | **~590€ /ml slurry** ~29.500€ / 50 ml slurry |
| **commercial 2** | GFP-Catcher Agarose | | | **~167€ /ml slurry** ~8.350€ / 50 ml slurry |

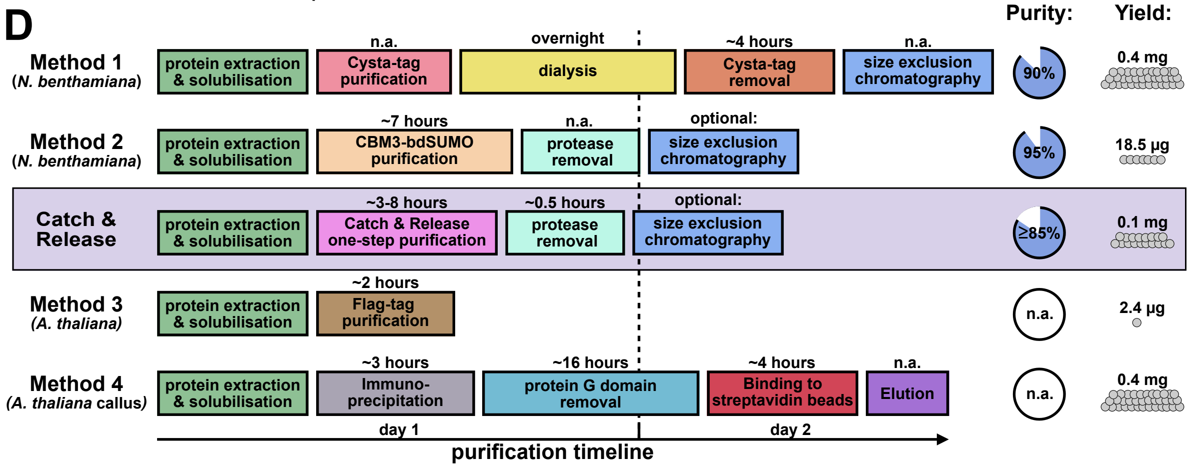


**Figure S3. Preparation, stability and cost-effectiveness of the homemade GFP-trap.**

(**A**) Step-by-step preparation of the homemade GFP-trap resin. Sepharose 4 Fast Flow resin was activated (2x 1 mM HCl; 1x PBS) and conjugated with purified KKK-GFP-clamp via N-terminal amine coupling to a target density of 0.8 mg/ml settled resin. Residual active sites were blocked using Tris-HCl buffer, followed by sequential high-salt (1 M NaCl) and ethanol (20% v/v) washes. The final product was stored at 4°C as a 50% (v/v) slurry in PBS containing 20% (v/v) ethanol. (**B**) Long-term stability of the homemade GFP-trap. The GFP binding capacity was quantified for newly prepared, 2-year-old, and 5-year-old resin preparations with a theoretical binding capacity of 0.8 mg/ml of resin bed volume. Data are presented as mean ± SEM (n = 3). Statistical significance was determined via one-way ANOVA, with different letters indicating significantly different groups. (**C**) A comparative cost analysis between the homemade GFP-Trap resin and two commercially available equivalents: ChromoTek GFP-Trap Agarose (ProductID: AB_2631357) and antibodies-online GFP-Catcher (ProductID: ABIN5311508). The cost is presented per ml of affinity resin slurry. The production of 50 ml of the homemade slurry can be completed in three days, requiring access to an FPLC system equipped with a size exclusion and IMAC column. Alternatively, batch purification using Ni-NTA beads is possible. Note that prices may vary by region and change. (**D**) Benchmark of the "Catch & Release" purification protocol against established methods in *N. benthamiana* (Method 1 (Sainsbury *et al.*, 2016) purification of human α1-antitrypsin; Method 2 (Islam *et al.*, 2019) isolation of human interleukin-6) and *A. thaliana* (Method 3 (Book *et al.*, 2010) purification of the 26S proteasome; Method 4 (Jeong *et al.*, 2018) isolation of mCherry). Purities were assessed with different methods, such as stained gel-based estimation (Method 2), mass photometry (Catch & Release) and ELISA assays (Method 1). Yields are given in gram of purified protein per gram of plant material.
