# Supplementary material for: Catch & Release—rapid cost‐effective protein purification from plants using a DIY GFP‐Trap‐protease approach
Source: Plant J. 2025 Nov 12;124(3):e70544. doi: 10.1111/tpj.70544 (PMC12611452; doi:10.1111/tpj.70544)
Supplement: Supplementary file 4 — Table S1. List of primers used in the present study. [file TPJ-124-0-s004.pdf]

Table S1: Primers used in this study

primer name	sequence
**Primer overhangs**	
Restriction-ligation_fw1	tttactagtATG
Restriction-ligation_fw2	tttggatccATG
Restriction-ligation_rev	tttcccggg
Gibson_fw	ggcctggcgcgccactagtggatccATG
Gibson_3C_rev1	ggaacagaacttccagactcccggg
Gibson_TEV_rev2	ggaagtagaggttctcactcccggg
GoldenGate_fw	tttgaagacttgatggcATG
GoldenGate_rev	tttgaagactttccacc
**Genotyping**	
WT_kea1_fwd	ccctcaaactcctacaatttctatg
WT_kea1_rev	gcaattattgcagtaatagccactgc
tDNA_kea1_rev	tagcatctgaatttcataaccaatctcgatacac
WT_kea2_fwd	gttgctatcactggcataattgc
WT_kea2_rev	ggatcaatggacatgcccac
tDNA_kea2_rev	attttgccgatttcggaac
**KEA1 and PGDH3 constructs**	
Kea1_gDNA_fwd	tttagtggatccatggagtatgcgtctacttttcaaagg
Kea1_gDNA_rev	tttcccggggattacgactgtgcctccttcga
Pgdh3_cDNA_fwd	tttagtggatccatggcgacgtctctgaatctatc
Pgdh3_cDNA_rev	tttcccgggtagtttgaggaaaacaaactcttcaatgg
Pgdh3_Gibson_fwd	ggcctggcgcgccactagtggatccatggcgacgtctctgaatct
Pgdh3_Gibson_rev	ggaagtagaggttctcactcccgggtagtttgaggaaaacaaactc
Pgdh3_GoldenGate_fwd	tttgaagacttgatggcatggcgacgtctctgaatct
Pgdh3_GoldenGate_rev	tttgaagactttccacctagtttgaggaaaacaaactc
Pgdh3_colonyPCR_fwd	gtcgaataattactcttcgatttg
Pgdh3_colonyPCR_rev	gtttacgtcgccgtccag
**Catch & Release vector**	
pGII_UBQ_1	gaagtcgtggttggaacgacttctttttccacg
pGII_UBQ_2	gaagtcgttccaaccacgacttcaaagcaag
pGII_Flag_1	catgggcggaggatctgactacaaggacgacgatgacaagtaggagct
pGII_Flag_2	cctacttgtcatcgtcgtccttgtagtcagatcctccgcc
pGII_Strep_1	catgggtgggtccagcgcatggtcacatccgcagtttgaaaaaggtgg
pGII_Strep_2	accaccacctttttcaaactgcggatgtgaccatgcgctggacccacc
pGII_Strep_3	tggtagcggtggtggttcaggtggtagtgcttggagccatcctcagttcgagaaataggagct
pGII_Strep_4	cctatttctcgaactgaggatggctccaagcactaccacctgaaccaccaccgct
pGII_MYC_1	catgggcggaggatcttctgaacaaaagttgatttcagaagaagatctgtaggagct
pGII_MYC_2	cctacagatcttcttctgaaatcaacttttgttcagaagatcctccgcc
pGII_HA_1	catgggcggaggatcttacccatacgatgttccagattacgcttaggagct
pGII_HA_2	cctaagcgtaatctggaacatcgtatgggtaagatcctccgcc
pGII_mCherry_1	ttccaaggctccatggtgagcaagggc
pGII_mCherry_2	cagcccatcgttttcttctgcattac

pGII_mCherry_3	gtaatgcagaagaaaacgatgggctggg
pGII_mCherry_4	catcttcatcttcatatgagctcctacttgtacagc
pGII_FAST_1	acgttatcactaaatggagcaacctac
pGII_FAST_2	aagctttataatgtcgcggaac
pGII_FAST_3	gcgacattataaagcttcgacgagtc
pGII_FAST_4	gatatattgtggtgtaacgttatcactaaatg
pGII_FRed_1	agtagtgtgctggccaccacg
pGII_FRed_2	tgagcttaccccactgatgtcat
pGII_FGreen_1	atcagtggggtaagctcacttgtacagctcgtc
pGII_FGreen_2	ggccagcacactactatggtgagcaaggg
pGII_3C_1	ccgggagtctggaagttctgttccaggggccctc
pGII_3C_2	catggagggcccctggaacagaacttccagactc
