# Supplementary material for: Catch & Release—rapid cost‐effective protein purification from plants using a DIY GFP‐Trap‐protease approach
Source: Plant J. 2025 Nov 12;124(3):e70544. doi: 10.1111/tpj.70544 (PMC12611452; doi:10.1111/tpj.70544)
Supplement: Supplementary file 5 — Table S2. List of vectors used in this study. [file TPJ-124-0-s007.pdf]

Table S2: Vectors used in this study

Plasmid Name	Source	Identifier
pG20_KEA1_TEV_mVenus_FR	Addgene	244953
pG20_KEA1_3C_mVenus_FR	Addgene	244954
pG20_KEA1_TEV_mVenus_FG	Addgene	244955
pG20_KEA1_TEV_mCherry_FR	Addgene	244956
pG20_KEA1_mCherry_TEV_mVenus_FR	Addgene	244957
pG20_KEA1_TEV_Strep_FR	Addgene	244958
pG20_KEA1_TEV_Flag_FR	Addgene	244959
pG20_KEA1_TEV_MYC_FR	Addgene	244960
pG20_KEA1_TEV_HA_FR	Addgene	244961
pG20_PGDH3_TEV_mVenus_FR	Addgene	244962
pG20_PGDH3_3C_mVenus_FR	Addgene	244963
pG20_PGDH3_mCherry_TEV_mVenus_FR	Addgene	244964
pG20_Venus_Hyg	Addgene	159703
pG20_mCherry_Hyg	Addgene	159701
pET28-MBP-super TEV protease	Addgene	171782
pet-NT*-HRV3CP	Addgene	162795
