# Supplementary material for: Catch & Release—rapid cost‐effective protein purification from plants using a DIY GFP‐Trap‐protease approach
Source: Plant J. 2025 Nov 12;124(3):e70544. doi: 10.1111/tpj.70544 (PMC12611452; doi:10.1111/tpj.70544)
Supplement: Supplementary file 6 — Table S3. Antibodies, bacterial strains, chemicals, software, and plant lines used in this study. [file TPJ-124-0-s008.pdf]

Table S3: Reagents and resources used in this study

REAGENT or RESOURCE	SOURCE	IDENTIFIER
**Antibodies**		
α-GFP	Roche	Prod. #: 11814460001
α-mCherry	Abcam	Prod. #: AB213511
α-Strep	Agrisera	Prod. #: AS21 4682
α-Flag	Sigma-Aldrich	Prod. #: A8592-.2MG
α-MYC	Agrisera	Prod. #: AS21 4685
α-HA	Agrisera	Prod. #: AS18 4176
**Bacterial and virus strains**		
Agrobacterium tumefaciens GV3101 competent cells (Hellens *et al.*, 2000)		N/A
Escherichia coli Bl21 (DE3) competent cells	ThermoFisher	Prod. #: EC0114
Escherichia coli DH5α competent cells	ThermoFisher	Prod. #: EC0112
**Chemicals, peptides, and recombinant proteins**		
Cellulase “Onozuka R-10”	Serva	Cat. No. / ID: 16419
Macerozyme R-10	Serva	Cat. No. / ID: 28302
Deoxynucleotide (dNTP) solution mix	New England Biolabs	Cat. No. / ID: N0447L
T5 Exonuclease	New England Biolabs	Cat. No. / ID: M0663L
Q5 High-Fidelity DNA Polymerase	New England Biolabs	Cat. No. / ID: M0491L

Taq DNA Ligase	New England Biolabs	Cat. No. / ID: M0208L
ß-Nicotinamide adenine dinucleotide (NAD^+)	New England Biolabs	Cat. No. / ID: B9007S
**Experimental models: Organisms/strains**		
Arabidopsis thaliana, Columbia-0, CS70000	European Arabidopsis Stock Centre (NASC)	NASC ID: N70000
Arabidopsis (Col-0) *kea1-1kea2-1* (Kunz *et al.*, 2014)		N/A
Arabidopsis (Col-0) *kea1-1kea2-1* pUBQ10:KEA1-TEV-mVenus_FAST-Red	This study	N/A
Arabidopsis (Col-0) *kea1-1kea2-1* pUBQ10:KEA1-3C-mVenus_FAST-Red	This study	N/A
Arabidopsis (Col-0) *kea1-1kea2-1* pUBQ10:KEA1-TEV-mVenus_FAST-Green	This study	N/A
Arabidopsis (Col-0) *kea1-1kea2-1* pUBQ10:KEA1-TEV-mCherry_FAST-Red	This study	N/A
Arabidopsis (Col-0) *kea1-1kea2-1* pUBQ10:KEA1-mCherry-TEV-mVenus_FAST-Red	This study	N/A
**Oligonucleotides**		
all oligonucleotides used in this study are listed in table S1	N/A	N/A
**Recombinant DNA**		
all vectors used in this study are listed in table S2	N/A	N/A
**Software and algorithms**		
GraphPad Prism (v10.3.0)	N/A	https://www.graphpad.com/features

ChimeraX (v1.7.1) (Meng *et al.*, 2023)		https://www.cgl.ucsf.edu/chimerax/
Fiji (Schindelin *et al.*, 2012)		https://imagej.net/software/fiji/

Hellens, R.P., Edwards, E.A., Leyland, N.R., Bean, S. and Mullineaux, P.M. (2000) pGreen: a versatile and flexible binary Ti vector for Agrobacterium-mediated plant transformation. *Plant Mol Biol*, **42**, 819-832. https://doi.org/10.1023/a:1006496308160

Kunz, H.-H., Gierth, M., Herdean, A., Satoh-Cruz, M., Kramer, D.M., Spetea, C. and Schroeder, J.I. (2014) Plastidial transporters KEA1, -2, and -3 are essential for chloroplast osmoregulation, integrity, and pH regulation in Arabidopsis. *Proceedings of the National Academy of Sciences*, **111**, 7480-7485. https://doi.org/10.1073/pnas.1323899111

Meng, E.C., Goddard, T.D., Pettersen, E.F., Couch, G.S., Pearson, Z.J., Morris, J.H. and Ferrin, T.E. (2023) UCSF ChimeraX: Tools for structure building and analysis. *Protein Science*, **32**, e4792. https://doi.org/10.1002/pro.4792

Schindelin, J., Arganda-Carreras, I., Frise, E., Kaynig, V., Longair, M., Pietzsch, T., Preibisch, S., Rueden, C., Saalfeld, S., Schmid, B., Tinevez, J.-Y., White, D.J., Hartenstein, V., Eliceiri, K., Tomancak, P. and Cardona, A. (2012) Fiji: an open-source platform for biological-image analysis. *Nature Methods*, **9**, 676-682. https://doi.org/10.1038/nmeth.2019
