# Supplementary material for: Catch & Release—rapid cost‐effective protein purification from plants using a DIY GFP‐Trap‐protease approach
Source: Plant J. 2025 Nov 12;124(3):e70544. doi: 10.1111/tpj.70544 (PMC12611452; doi:10.1111/tpj.70544)
Supplement: Supplementary file 8 — Data S1. Amino acid sequence of GFP‐clamp including N‐terminal tags. [file TPJ-124-0-s003.pdf]

>His8-StrepII-Trx-KKK-GFPc

MKHHHHHHHHGASWSHPQFEKGGGSDKIIHLTDDSFDTDVLKADGAILVDFWAEWCGPCKMIAPILDEIADEYQ
GKLTVAKLNIDQNPGTAPKYGIRGIPTLLLFKNGEVAATKVGALSKGQLKEFLDANLAGSGSGENLYFQGAMGKKK
GSDLGRMLLEAARAGQDDEVRILMANGADVNAADDVGVTPLHLAAQRGHLEIVEVLLRYGADVNAADLWGQTP
LHLAATAGHLEIVEVLLRNGADVNARDNIGHTPLHLAAWAGHLEIVEVLLRYGADVNAQDRFGHTPFDLAIDNGNE
DIAEVLQRAAGGGSGGGDVNAYDEVGWTPLHRAAWGHLELVERLLRNGADVNAADIDGYTPLHLAAFSGHLEIV
EVLLRYGADVNADDQAGFTPLHLAAIFGHLEIVEVLLRNGADVNAQDRFGHTPFDLAIDNGNEDIAEVLQRAA*
